# Developing and evaluating a situated psychometric instrument for assessing climate anxiety: The SAM^2^ CAM

**DOI:** 10.1111/aphw.70125

**Published:** 2026-02-04

**Authors:** Chiara. K. V. Hill‐Harding, Marissa. D. Klein, Constantin von Stackelberg, Esther. K. Papies, Lawrence. W. Barsalou

**Affiliations:** ^1^ School of Psychology & Neuroscience University of Glasgow UK; ^2^ Behavioural Science Institute Radboud University Netherlands

**Keywords:** climate anxiety, climate change, eco‐anxiety, individual differences, mental health, situated assessment method

## Abstract

Although increasing research examines climate anxiety, little is known about the situational factors related to it. To assess these factors, we developed and evaluated a situated psychometric instrument for assessing how much climate anxiety individuals recall experiencing in 31 situations where climate anxiety is likely (e.g., hearing about climate catastrophes on the news). Of interest was how climate anxiety is experienced in a country like the UK, where climate disasters are mostly heard about in the media and anticipated in the future, relative to countries where climate disasters are experienced directly and regularly. In an online survey (*N* = 303; 50.8% female), we investigated how much climate anxiety individuals recall experiencing in situations where climate anxiety is likely to occur, along with how much they recall experiencing 13 factors potentially related to climate anxiety (e.g., threat, violation, rumination). An individual measure of climate anxiety, averaged across situations, exhibited high reliability, construct validity and content validity. Climate anxiety varied widely across situations, with individuals further varying in how much climate anxiety they remembered experiencing in each situation. As predicted, the 13 situational factors tended to correlate significantly with climate anxiety across situations, explaining a median 75% of its variance in individual regressions.

## INTRODUCTION

In response to threats posed by climate change, people increasingly experience diverse forms of climate anxiety that potentially challenge their mental health and well‐being. Here we define climate anxiety as anxiety specific to anthropogenic climate change and its experienced or anticipated consequences for ecosystems, biodiversity and human life (Pihkala, 2020). Climate anxiety has many dimensions and is often related to worry and fear, although it is expressed as a distinct construct (Pihkala, 2020). Climate anxiety can also be understood as an existential anxiety that challenges humans' core existential experiences, including happiness, identity, meaning, death, freedom and isolation (Passmore et al., 2023). It can arise from direct impacts, such as physical experience with climate change, from indirect impacts such as climate change impacting politics and local weather, or from overarching impacts such as knowledge, education and awareness of climate change, which can come from vicarious encounters, including media reports and anticipated threats (e.g., Clayton, 2020; Maran & Begotti, 2021; Pihkala, 2020). Hence, anyone can be affected by climate anxiety as a result of diverse influencing factors (Clayton, 2020).

While extremely high levels of climate anxiety can impact a person's functioning and wellbeing, climate anxiety is not in itself pathological, and can be viewed as a rational response to a realistic threat (Clayton, 2020; Pihkala, 2020). This distinguishes climate anxiety from classic anxiety disorders, meaning that it cannot be entirely explained by general trait anxiety (Pihkala, 2020). From theoretical proposals, it appears that uncertainty, uncontrollability and unpredictability associated with future climatic changes play a major role in climate anxiety (Pihkala, 2020).

As a result of these factors, populations in countries like the UK can experience climate anxiety, even though their perhaps most common current concerns are anticipating significantly worse consequences in the future. Unsurprisingly, populations in high‐risk countries (e.g., Nigeria, Chad, Somalia, and Syria) who already experience extreme consequences of climate change tend to experience higher climate anxiety than populations in lower‐risk countries, with serious mental health consequences (e.g., Cunsolo & Ellis, 2018; Ojala et al., 2021; Stanley et al., 2021). Furthermore, factors such as being female, younger, and having less education are linked to increased risk of climate anxiety in lower‐risk countries (Poortinga et al., 2019). Finally, a variety of other factors, such as national politics (Poortinga et al., 2019), family contexts (Lawson et al., 2019) and local temperature spikes (e.g., Sugerman et al., 2021), have been found to be associated with an individual's climate anxiety and concern.

In the work reported here, we developed and evaluated a climate anxiety instrument for the type of climate anxiety typically experienced in countries like the UK, where this study took place. Of particular interest was assessing trait levels of climate anxiety across situations where individuals report it. Also of interest was establishing situations where climate anxiety is highest, along with situational factors associated with climate anxiety. Although we assume that an individual's climate anxiety varies considerably across situations, we nevertheless assume that stable differences exist between individuals in their average trait‐levels of climate anxiety across situations.

### Climate anxiety as a constructed emotion

Some theoretical perspectives on climate anxiety suggest that it is highly malleable, varying widely between individuals and even within them (Pihkala, 2020). Because experiences of climate anxiety result from a wide variety of environmental and social factors, it can vary as diverse factors continually contribute to the specific forms it takes (e.g., local climate disasters, media communication about climate disasters, new social norms, changing weather patterns, silence around climate change; Pihkala, 2020).

The constructivist perspective of emotion offers a potentially powerful way of understanding how climate anxiety takes different forms both between and within individuals (L. Feldman Barrett, 2006; L. Feldman Barrett, 2017; L. F. Feldman Barrett, 2006; Gendron & Barrett, 2009; Lebois et al., 2020; Wilson‐Mendenhall et al., 2011). From this theoretical perspective, an emotion is a constructed category that depends heavily on an individual's culture, language and learned conceptualisations of experience—similar to how categories for common objects, such as tools and furniture, are acquired. Rather than being implemented by an innate circuit in the brain, an emotion category is acquired throughout lifespan development, taking infinitely many forms as it is expressed in daily life. From this theoretical perspective, fear, for example, is viewed as an acquired category that takes diverse forms across situations, such as concern, alarm, fright, dread and terror. Certainly, genetically based biological systems contribute to emotion categories (Lebois et al., 2020; LeDoux, 2012), as they do to all categories (e.g., the extraction and processing of perceptual features in plant and animal categories; Malt, 1995). Nevertheless, a wide variety of learning, social, linguistic and cultural systems contribute to the experience and conceptualisation of emotion as well. As a result, each individual's emotion categories take specific forms that reflect their unique biology and situational experience across the lifespan.

From the constructivist perspective, anxiety can be viewed as a constructed emotion category, with climate anxiety emerging as a sub‐category in those individuals for whom it becomes relevant. For individuals who have so far avoided climate disasters or refused to acknowledge them, climate anxiety may not emerge as an emotion category that plays a central role in their experiences. For other individuals, however, climate anxiety may emerge as an emotion category that becomes highly salient on a daily basis. Because climate anxiety is a constructed category that reflects each individual's unique experience, it takes diverse forms across the individuals who do experience it. For individuals who have experienced climate disasters directly, climate anxiety takes forms that reflect these situational features of their experience. For individuals who only anticipate climate disasters and experience them indirectly in the media, climate anxiety likely takes different forms.

### Psychometric assessment of climate anxiety

For the reasons just presented, we assume here that climate anxiety is a constructed emotion. From this perspective, a psychometric instrument is needed that captures the unique forms of climate anxiety in different individuals, reflecting each person's unique situational experience. As will be seen, we believe that current psychometric instruments cannot fully capture an individual's climate anxiety because they only assess the individual abstractly, not with respect to specific situations.

To date, climate anxiety (e.g., Clayton & Karazsia, 2020; Hogg et al., 2021), along with related concepts such as climate worry (e.g., Stewart, 2021) and climate emotions (e.g., Contreras et al., 2024), has typically been assessed psychometrically in a non‐situated manner at the individual trait level, even when measuring climate anxiety in daily assessment studies (e.g., Contreras et al., 2024). To do so, existing measures assess climate anxiety or climate worry with general statements about the individual that ignore specific situational experience, such as “I worry that I might not be able to cope with climate change” (Stewart, 2021) or “I find myself crying because of climate change” (Clayton & Karazsia, 2020). Items like these do not reference specific situations but instead ask about individual experience abstracted across situations. Averaging across a set of unsituated items establishes a trait‐level measure for an individual that is decontextualised, not assessing the construct of interest for the situations where it is experienced (Dutriaux et al., 2023).

Unsituated approaches like these assume that individuals can abstract across diverse situations that are potentially relevant to the items being assessed. From a cognitive perspective, it is an open question whether people actually attempt to abstract across all relevant situations, and if so, whether they do so accurately (Dutriaux et al., 2023). Perhaps more realistic accounts of how people produce responses to unsituated assessment items are that they use the availability strategy to sample a small subset of situations that are currently available in memory (Tversky & Kahneman, 1973) and/or that they consult intuitive theories about climate anxiety and its relation to their self‐concepts (Gelman & Legare, 2011; Nisbett & Wilson, 1977; Ross, 1977).

Finally, unsituated approaches make the implicit assumption that climate anxiety is relatively constant across relevant situations, which is unlikely (Pihkala, 2022). Indeed, much research finds that an individual's experiences and behaviours depend more on situational factors than on individual differences (e.g., Bandura, 1978; Cervone, 2005; Fleeson & Jayawickreme, 2021; Mischel, 1968; Mischel & Shoda, 1995). Not only do traits vary extensively across situations, but individuals vary widely in how they express traits in the same situations (i.e., individual by situation interactions; Dutriaux et al., 2023). It follows that climate anxiety cannot be captured entirely by a single trait‐level measure (Pihkala, 2020). As Pihkala (2022) notes, individual variability and experiences across different situations influence emotions like climate anxiety substantially. Assessing climate anxiety at both the individual and situational levels may thus provide a much richer assessment than only measuring it at the individual level.

### Developing a situated measure of climate anxiety

As described earlier, we assume that climate anxiety is a constructed emotion, with different individuals constructing different forms that reflect their unique situational experience. To capture and understand the unique form of climate anxiety that each individual constructs, it is therefore essential to assess the situations where they are likely to experience it. A situated assessment not only establishes each individual's trait level of climate anxiety (their average climate anxiety across situations) but also establishes the situations where an individual is most and least likely to experience climate anxiety (the vector of climate anxiety as it varies across situations). Once a situational profile of climate anxiety has been established for an individual, we know the situations most likely to induce it in their life and can therefore work with these specific situations to reduce it (if desired).

Originating in basic research on situated cognition (e.g., Barsalou, 2008; Newen et al., 2018; Robbins & Ayede, 2009), the Situated Assessment Method (SAM^2^) provides a novel framework for assessing individual differences in constructs on two dimensions of situatedness: situational experience and the Situated Action Cycle. Specifically, to measure a particular construct, a SAM^2^ instrument assesses it, first, in situations where it occurs, and second, across phases of the Situated Action Cycle (i.e., where: (1) events in the *environment* (2) trigger cognitive states of *self‐relevance*, which in turn (3) induce *affect*, (4) initiate *action* and (5) produce *outcomes*; Barsalou, 2020). Rather than measuring a construct in an abstract decontextualised manner, a SAM^2^ instrument assesses it embedded in these two dimensions of situatedness (referred to by the superscript ^2^ in SAM^2^). Besides establishing a trait‐level measure of an individual across situations, a SAM^2^ instrument further establishes a unique profile of how each individual experiences the construct across situations (typically varying considerably), along with how the construct relates to other constructs in the Situated Action Cycle (establishing construct validity). For a detailed discussion of these points, specifically, and of the SAM^2^ approach in general, see Dutriaux et al. (2023). To date, the SAM^2^ approach has been applied effectively to constructs associated with behaviour (e.g., healthy and unhealthy habits; Dutriaux et al., 2023), physical health (e.g., water drinking; Roger et al., 2024) and mental health (e.g., trichotillomania; Taylor Browne Lūka et al., 2024).

### Overview

We first developed a situated psychometric instrument for assessing climate anxiety—the Situated Assessment Method for Climate Anxiety Measurement (SAM^2^ CAM)—and then used it to evaluate UK adults' climate anxiety across 31 everyday situations. Specifically, the SAM^2^ CAM assesses how much climate anxiety individuals recall experiencing previously in 31 situations where climate anxiety is likely to occur. Of interest was how climate anxiety is experienced in a country like the UK, where climate disasters are mostly heard about in the media and anticipated in the future, relative to countries where climate disasters are experienced directly and regularly.

Informed by previous literature, we sampled these situations from four domains likely to be relevant to climate anxiety in Western populations like the UK, reflecting indirect, overarching and less severe direct climate change impacts: (1) media exposure (Luo & Zhao, 2021; Maran & Begotti, 2021; Ogunbode et al., 2020; Usher et al., 2019), (2) experiencing climate change or environmental pollution (Dodd et al., 2018; Hrabok et al., 2020), (3) social interactions related to climate change (Galway et al., 2021; Hickman et al., 2021; Hoggett & Randall, 2018) and (4) performing (un)sustainable habitual behaviours (Galway et al., 2021; Godden et al., 2021; Lawson et al., 2019; Verplanken & Roy, 2013). Table 1 presents these 31 situations. Following the SAM^2^ approach, we assessed these 31 situations on climate anxiety (dependent variable) and on 13 situational factors (predictors) from the Situated Action Cycle likely to influence climate anxiety in these situations (cf. Barsalou, 2020; Dutriaux et al., 2023). Table 2 presents the specific scales used to assess the dependent measure and its 13 predictors.

**TABLE 1:** The 31 situations assessed in the SAM^2^ CAM.

Abbreviation	Situation
S01 – Catastrophes	Hearing about climate catastrophes on the news (e.g., flooding, wildfires)
S02 – Unsustainable ads	Seeing ads for animal products or single‐use items.
S03 – Activism news	Hearing about climate activism on the news.
S04 – Government's plan	Hearing news about the government's climate action plan not being achieved.
S05 – Documentaries	Watching animal/nature documentaries.
S06 – Influencers	Seeing influencers promote environmentally unsustainable lifestyles on social media (e.g., using private jets, ‘fashion hauls’).
S07 – Green energy	Seeing news on advances in green energy production.
S08 – Local product ads	Seeing ads for locally sourced products.
S09 – Litter	Seeing litter in the street.
S10 – Air pollution	Perceiving air pollution caused by fossil fuels (e.g., when a car drives by).
S11 – Left‐overs	Seeing someone throw away food left‐overs.
S12 – Water running	Seeing someone leave the water running.
S13 – Car running	Seeing someone leave their car running while parked.
S14 – Communal action	Seeing pro‐environmental action in your community (e.g., a school planting trees).
S15 – Denier talk	Talking about climate change to someone who does not believe in it.
S16 – ‘Green person’ talk	Talking with someone who is giving up some kind of pleasure to be ‘green’ (e.g., giving up eating meat).
S17 – Take‐away	Ordering take‐away that is delivered in non‐recyclable containers.
S18 – Taking car	Taking the car when I have a choice not to.
S19 – Disposable cups	Using disposable cups when I have a choice not to.
S20 – Plastic wrapping	Purchasing items wrapped in plastic.
S21 – Plant‐based food	Choosing to eat plant‐based products instead of animal products.
S22 – Reusable hygiene	Buying reusable hygiene and cleaning products (e.g., washable sponges).
S23 – Children's future	Thinking about the future that children in the current generation may experience.
S24 – Second‐hand	Buying clothes second‐hand.
S25 – Too much food	Purchasing more food than intended.
S26 – Aeroplane	Travelling by aeroplane.
S27 – Recycling	Recycling according to guidelines.
S28 – Reusable bags	Bringing reusable bags when shopping.
S29 – Climate activism	Engaging in climate activism (e.g., attending protests, sharing posts online).
S30 – Nature	Visiting a loved place in nature.
S31 – Own future	Thinking about my future.

**TABLE 2:** Judgement scales grouped by phase of the situated action cycle (SAC), with their intraclass correlations.

SAC phase	Judgement name/query/values/labels	ICC2	ICC3	ICC3k
Affect	**Anxiety**			
	When this situation occurs, how much anxiety do you experience about climate change?	.21	.44	.96
	(0 to 10)/ (no anxiety at all, moderate anxiety, extreme anxiety)			
Environment	**Frequency**			
	How frequently do you experience each of the following situations?	.38	.17	.87
	(0 to 10)/ (never, once a month, multiple times a day)			
Environment	**Concern**			
	How concerned are other people in this situation about climate change and sustainability?	.11	.38	.95
	(0 to 10)/ (not concerned at all, moderately concerned, extremely concerned)			
Self‐relevance	**Violation**			
	How much does this situation violate your expectations?	.28	.28	.92
	(0 to 10)/ (no violation at all, moderate violation, extreme violation)			
Self‐relevance	**Threat**			
	How threatened do you feel by what happens in this situation?	.26	.39	.95
	(0 to 10)/ (not threatened at all, moderately threatened, extremely threatened)			
Self‐relevance	**Opportunity**			
	How much of an opportunity do you see in this situation to do something constructive?	.21	.29	.93
	(0 to 10)/ (no opportunity at all, moderate opportunity, extremely good opportunity)			
Affect	**Habits motivation**			
	How much does being in this situation motivate you to adopt sustainable habits?	.11	.42	.96
	(0 to 10)/ (not at all, moderately, extremely)			
Affect	**Action motivation**			
	How much does being in this situation motivate you to work on social action to promote sustainability?	.05	.63	.98
	(0 to 10)/ (not at all, moderately, extremely)			
Action	**Control**			
	How much control do you believe you have over what happens in this situation?	.45	.20	.88
	(0 to 10)/ (no control at all, moderate control, full control)			
Action	**Coping**			
	How effectively are you able to cope with this situation	.18	.34	.94
	(0 to 10)/ (not effective at all, moderately effective, extremely effective)			
Action	**Rumination**			
	How likely are you to ruminate about this situation?	.11	.44	.96
	(0 to 10)/ (not likely at all, somewhat likely, extremely likely)			
Action	**Compassion**			
	How judgmental/compassionate are you about yourself and other people in this situation?	.41	.08	.73
	(−5 to 5)/ (extremely judgemental, neither judgemental nor compassionate, extremely compassionate)			
Outcome	**Consequences**			
	How much do you think about the consequences of what happens in this situation for climate change and sustainability?	.07	.41	.96
	(0 to 10)/ (not at all, somewhat, a lot)			
Outcome	**Disruption**			
	How much does this situation disrupt your life?	.10	.40	.95
	(0 to 10)/ (not at all, moderately, extremely)			

*Note*: The left column shows the phase of the Situated Action Cycle from which each measure was sampled. The first row presents the climate anxiety measure used in the SAM^2^ CAM, followed by the measures for 13 SAM2 predictors (each preceded by its name in bold). Below each measure are the end points of the continuous slider scale used to assess it, together with the scale's labels. The right columns contain three measures of agreement and reliability (using the names for them from Shrout & Fleiss, 1979: ICC2, ICC3 and ICC3k). Specifically, ICC2 is the interrater agreement for each measure across the 31 situations, assessing how much participants agree in evaluating the 31 situations on a specific measure (with participants treated as random effects). The ICC3 is the coherence of the 31 situations as test items on the SAM^2^ CAM, namely, how much they agree in ordering participants from highest to lowest on the respective measure (with the situations treated as fixed effects). The ICC3k is Cronbach's alpha for the overall score of each measure (aggregated across the 31 situations serving as test items for each participant), capturing the measure's test reliability in ordering participants from highest to lowest.

Nine of the predictors assessed in the SAM^2^ CAM have previously exhibited a positive relationship with climate anxiety: event *frequency* (Clayton & Karazsia, 2020; Maran & Begotti, 2021; Usher et al., 2019), *threat* (e.g., Soutar & Wand, 2022; Stollberg & Jonas, 2021), *rumination* (Clayton & Karazsia, 2020; Hogg et al., 2021), expectation *violation* (Dutriaux et al., 2023), anticipated *consequences* (Farrokhi et al., 2020; Myers, 2014), perceived *opportunity* to take constructive action (Maran & Begotti, 2021), *motivation* for sustainable *habits*/behaviours (Clayton, 2020; Verplanken & Roy, 2013), *motivation* for social climate *action* (Bright & Eames, 2022; Stanley et al., 2021) and *disruption* to one's life (Ágoston et al., 2022; Brulle & Norgaard, 2019). We therefore expected these predictors to correlate positively with climate anxiety. Conversely, four additional factors have previously exhibited a negative relationship with climate anxiety: *control* (Cunsolo & Ellis, 2018; Grupe & Nitschke, 2013; Pihkala, 2020), other people's (perceived) *concern* (Norgaard, 2011; Pihkala, 2018), *coping* abilities (Ogunbode et al., 2019; Ojala, 2013) and self‐*compassion* (Gerber, 2023). We hence expected these predictors to correlate negatively with climate anxiety. Because all 13 predictors have been associated with climate anxiety in previous literature, we assumed that they would be similarly associated with it across the 31 situations here.

To evaluate the SAM^2^ CAM, we developed the following hypotheses prior to data collection and then assessed them in a UK sample. Because the hypotheses in the original version of this article (pre‐print Hill‐Harding et al., 2024) did not include specific statistical benchmarks, these were added following pre‐print publication based on data in previous articles, thereby reflecting standard values obtained when applying the SAM^2^ approach (see, e.g., Dutriaux et al., 2023; Roger et al., 2024; Taylor Browne Lūka et al., 2024).

**Large reliable individual differences in participants' climate anxiety.** Individuals would reliably exhibit considerable variability in their trait‐levels of climate anxiety, averaged across the 31 SAM^2^ CAM situations. Specifically, we expected that mean individual scores for climate anxiety across the 31 situations would range across at least half the scale from 2.5 to 7.5 and exhibit test reliability of at least .80.

**Substantial situational effects**. Situations would exhibit considerable variability in their average level of climate anxiety across individuals. Specifically, we expected that a given participant would report having previously experienced high climate anxiety in some situations but low climate anxiety in others, such that their judgments for specific situations typically range across the entire scale from 0 to 10.

**Substantial situation by individual interaction.** A large situation by individual interaction would emerge for climate anxiety (i.e., the pattern of climate anxiety across the 31 situations would vary considerably across different individuals), with the intraclass correlation for individual agreement across situations being less than .40.

**High construct validity for climate anxiety with large individual differences in prediction profiles.** If the SAM^2^ CAM measure for climate anxiety has construct validity, it should correlate moderately to highly with related constructs. We therefore predicted that climate anxiety should correlate with each of the 13 predictors from the Situated Action Cycle in its predicted positive or negative direction (>|.30|). Additionally, we predicted that the prediction profiles for different individuals would vary widely across the 13 predictors.

**High content validity for climate anxiety.** If the SAM^2^ CAM measure for climate anxiety has content validity, the 13 predictors should explain its variance comprehensively. We therefore predicted that the 13 predictors would typically explain at least 60% of the variance in an individual's climate anxiety.

***E1*. Exploratory question:** We explored correlational relations of climate anxiety with general anxiety, stress and depression. Because the measures for general anxiety, stress and depression were all unsituated, only modest correlations between them and the SAM^2^ CAM were likely to emerge, reflecting shared variance at the individual trait level. Because the SAM^2^ CAM also captures variance related to a specific set of situations, it is likely to diverge significantly from measures that aren't situated, thereby resulting in weak correlations between them. Finally, as described earlier, climate anxiety may often not be closely related to general anxiety, further suggesting a weak relationship between them.

## METHODS

### Design

This study was part of a larger project that collected data from a common set of participants for three related studies on climate change. For this study, all participants responded to the SAM^2^ CAM's 31 situations (Table 1), first, for climate anxiety (dependent variable) and then for the 13 predictors from the Situated Action Cycle (frequency, concern, violation, threat, opportunity, habits motivation, action motivation, control, coping, rumination, compassion, consequences and disruption; Table 2). Using the 21‐item version of the Depression Anxiety Stress Scale (DASS‐21; Lovibond & Lovibond, 1995), three additional non‐situated measures were collected for each participant: general anxiety, depression and stress. To avoid issues with missing data, participants were required to answer all questions (forced response format).

### Participants

A sample of 330 UK residents was sampled from the Prolific recruitment platform (prolific.co), requiring that they were fluent in English, aged 18 to 79, and had completed at least 10 previous Prolific studies with at least a 95% approval rate. The participants included in the study were individuals interested in participating and volunteered for pay, not participants we selected. Because we were interested in how climate anxiety is experienced by individuals in a relatively protected county, this sample was appropriate, given that UK residents have likely been exposed to more vicarious encounters (e.g., news) than to personal experiences with climate change.

Reflecting the availability of funding, participants were recruited over three data collection periods in April and July 2022. Participants were excluded if they exhibited repetitive responding (flatlining) and/or random responding across two or more of the measures across any of the three studies associated with the larger project. This resulted in the removal of 27 participants, leaving a final sample of *N* = 303. We determined this sample size to be sufficient, based on Schönbrodt and Perugini's (2013) recommendation that a sample size of 250 estimates population values of correlations accurately. Table 3 presents the demographic features of the final sample. As can be seen, the sample's demographic features correspond closely to those of the general UK population.

**TABLE 3:** Participant demographics.

Sample demographic characteristic	Sample	UK population
Gender		
Male	48.8%	49.0%
Female	50.8%	51.0%
Non‐binary	0.3%	‐‐
Country of residence		
England	83.8%	84.3%
Scotland	9.2%	‐‐
Wales	3.6%	‐‐
Northern Ireland	3.0%	‐‐
Other	0.3%	‐‐
Ethnicity		
White	89.4%	81.7%
Asian	5.0%	9.2%
Black	3.0%	4.0%
Other/mixed	2.6%	2.9%
Median age	41.0	40.7
Education (highest achieved)		
No qualification	2.7%	18.2%
Level 1 to 3 (GCSE/A‐level/high school diploma)	19.8%	39.9%
Some college but no degree	10.6%	‐‐
Trade/vocational training/apprenticeship	9.2%	8.1%
Level 4 + (bachelor's/master's/doctorate)	57.9%	33.8%

*Note*: Data on UK population based on 2021 UK census data from the UK Government website (https://www.ethnicity‐facts‐figures.service.gov.uk/ accessed 25th February 2025 and https://www.ons.gov.uk/peoplepopulationandcommunity for education data, accessed 19th December 2025).

### Materials

As described earlier, the situations (Table 1) and predictors (Table 2) used in the SAM^2^ CAM were established through existing literature on climate anxiety and the Situated Action Cycle (cf. Barsalou, 2008, 2020). Specifically, we sampled these situations from four domains likely to be relevant to climate anxiety in the UK, mainly reflecting indirect, overarching and less severe direct climate change impacts related to: (1) media exposure (Luo & Zhao, 2021; Maran & Begotti, 2021; Ogunbode et al., 2020; Usher et al., 2019); (2) experiencing climate change or environmental pollution (Dodd et al., 2018; Hrabok et al., 2020); (3) social interactions related to climate change (Galway et al., 2021; Hickman et al., 2021; Hoggett & Randall, 2018); (4) performing (un)sustainable habitual behaviours (Galway et al., 2021; Godden et al., 2021; Lawson et al., 2019; Verplanken & Roy, 2013). For a detailed overview of the selection process that determined the final situations included, please see SM‐7.

The 14 measures presented earlier in Table 2, including climate anxiety, were assessed for each of the 31 situations in Table 1 on a continuous 11‐point slider scale from 0 to 10, with 0.1 decimal accuracy. Table 2 presents the verbal anchors used for each scale. If a participant had not previously experienced a situation, we asked them to imagine being in it and then to evaluate their experience of the imagined situation (SM‐1 presents the full instructions). Much research shows that imagined situations have the same expected emotional impact as experiencing them and can thus be used as valid stimuli (Lench et al., 2011).

To additionally assess the relationships of climate anxiety with general anxiety, stress and depression, we included the DASS‐21 (Lovibond & Lovibond, 1995), which has satisfactory psychometric properties and is applicable across countries (Bibi et al., 2020; Le et al., 2017). All materials are publicly available online at: https://osf.io/2zg5u/overview%3Fview_only=e5f93c90da4a4c31b17ac189e7a2c642.

### Procedure

Participants were recruited online via Prolific (www.prolific.co, 24th February 2022) and then redirected to the Qualtrics survey (Qualtrics, Provo, UT, 2018). Participants were informed about the general purpose of the study, the study's procedure, payment details, their rights, data storage and how to contact us. Consent was given electronically by selecting “I consent” from a drop‐down menu. No personal information was obtained from participants, and all responses were stored securely. Participants were paid at a rate of £7.50 per hour. This study was approved by the University of Glasgow College of Science & Engineering's Ethics Committee.

Participants first responded to the DASS‐21 (Lovibond & Lovibond, 1995). They then received general instructions for responding to the SAM^2^ CAM, along with specific instructions for each SAM^2^ CAM measure just prior to evaluating it (provided in SM‐1). Participants next evaluated climate anxiety for the 31 situations, which were presented in a different random order for each participant. Thereafter, participants responded to the SAM^2^ CAM another 13 times for each predictor in Table 2. Participants received the predictor blocks in a fixed order that we thought would make intuitive sense to them (frequency, concern, violation, threat, opportunity, habits motivation, action motivation, control, coping, rumination, compassion, consequences and disruption). This seemed most appropriate, also given that Dutriaux et al. (2023) found that a fixed order of predictor blocks produced the same results as randomising them. Within each predictor block, however, the 31 situations were randomised for each participant. Participants performed each judgment using a slider scale that recorded responses with one decimal point accuracy. After completing the SAM^2^ CAM, participants were asked about their survey experiences, debriefed and given resources for help with anxiety. The median time to complete the study was 51 minutes (min = 22, max = 321).

All statistical analyses were performed in R (version 4.2.0; R Core Team, 2021), using R Studio. Linear regression analyses regressed climate anxiety onto the 13 predictors. Pearson correlations were computed to investigate the relationships between climate anxiety and each of the DASS‐21 subscales (Lovibond & Lovibond, 1995). The full set of R scripts with code and data sets are publicly available online https://osf.io/2zg5u/overview%3Fview_only=e5f93c90da4a4c31b17ac189e7a2c642.

## RESULTS

### Individual differences (H1)

**FIGURE 1:**
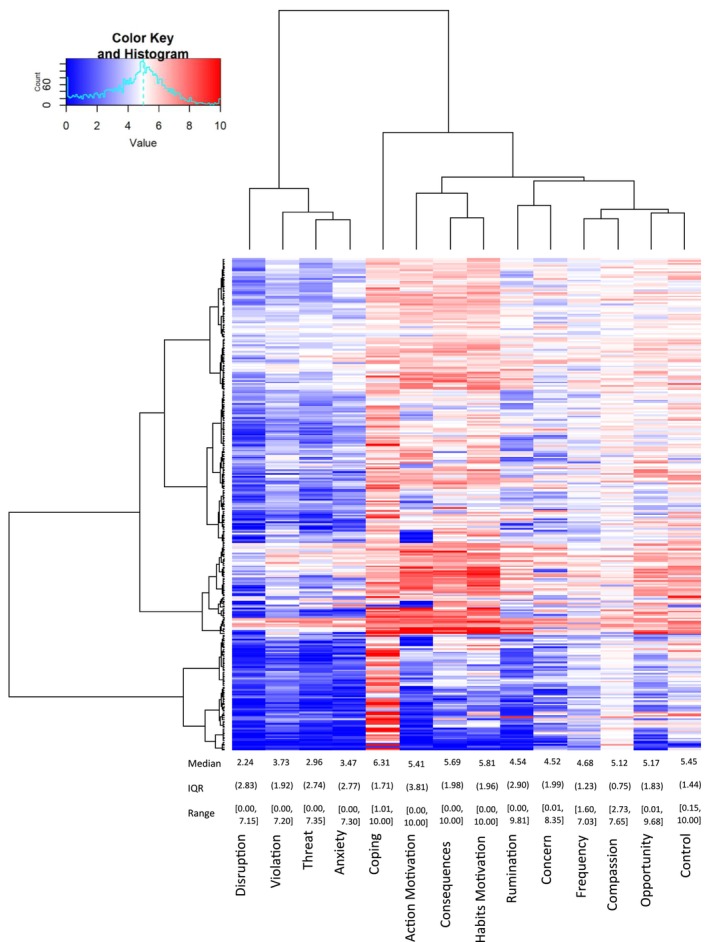
Visualisation of the judgement means for each participant across measures, with the median, IQR and range presented for each measure towards the bottom. *Note*: Each row contains the 14 mean judgements for one of the 303 participants across situations. As cells become redder, they represent higher mean judgements. As cells become bluer, they represent lower mean judgements. Because Compassion was rated from ‐5 to 5, it was put onto a 0 to 10 scale here to allow for comparisons with the other measures. The dendrogram on the left resulted from hierarchical clustering using the Ward D measure. Median shown for each measure, with IQR in round brackets, and range in square brackets.

We first computed an overall trait‐level measure of climate anxiety for each participant by averaging their climate anxiety judgments across the 31 situations (with a grand mean across participants and situations of *M* = 3.33, *SD* = 1.83, 95% CI [3.12, 3.53]). We then computed the analogous trait‐level averages for each of the 13 predictors. Figure 1 visualises the distributions of these individual measures, one measure per column, with each row representing the 14 mean judgments for a single participant. Overall measures of central tendency and variability appear at the bottom of the figure. As Figure 1 illustrates, clear individual differences emerged in climate anxiety scores, with a range of 7.30. Large individual differences similarly occurred for how frequently participants encountered the 31 situations and how much they experienced the 12 other predictors of climate anxiety. Interestingly, most measures tended to exhibit relatively moderate levels across participants (e.g., frequency, concern, action motivation, control and rumination). Only coping exhibited a relatively high level, indicating that participants felt they could cope well in most situations. Surprisingly, and in contrast to our expectations, anxiety, violation, threat and disruption all exhibited relatively low levels. For the table of raw situation means across measures, see SM‐2.

#### Exploratory observations

As the clustering solution on the left of Figure 1 illustrates, different groups of participants emerged from this analysis. The large cluster at the bottom exhibited the lowest levels of climate anxiety and related measures, along with the highest coping. In contrast, the next cluster up exhibited the highest anxiety and the lowest coping, with the clusters above exhibiting intermediate levels. As the clustering solution along the top illustrates, a relatively small cluster of measures exhibiting negative facets of climate anxiety emerged on the left with relatively low values. To the right, a larger cluster of measures emerged with relatively high values for coping, action, motivation and consequences. These two clusters suggest that participants experienced relatively little negative emotion for the 31 situations while experiencing relatively high levels of motivation and efficacy.

### Test reliability (H1)

To measure climate anxiety and individual differences reliably, the SAM^2^ CAM should exhibit high test reliability (e.g., Huscroft‐D'Angelo et al., 2020). We used Cronbach's alpha to measure how well the SAM^2^ CAM situations can reliably establish participants' overall trait‐level scores (averaged across the 31 situations that served as test items) on each of its 14 measures (Cronbach, 1951; ICC3k in Shrout & Fleiss, 1979). Intuitively, alpha estimates how reliably the same set of participants would exhibit the same average scores across the same situations as test items, if tested again under comparable conditions. Because we were only interested in the reliability of overall measures, coefficient alpha was sufficient for this purpose. Because it is not necessary that the *situations* in the SAM^2^ CAM exhibit internal consistency (Dutriaux et al., 2023), it was not appropriate to assess coefficient omega (Flora, 2020).

As Table 2 illustrates, almost all measures exhibited excellent test reliability above .90, and all were above an acceptable level of .70 (Cronbach, 1951; Warrens, 2017). Most significantly, the SAM^2^ CAM measure of climate anxiety exhibits a test reliability of .96.

### Situation profiles (H2a)

#### Variability in situation profiles

**FIGURE 2:**
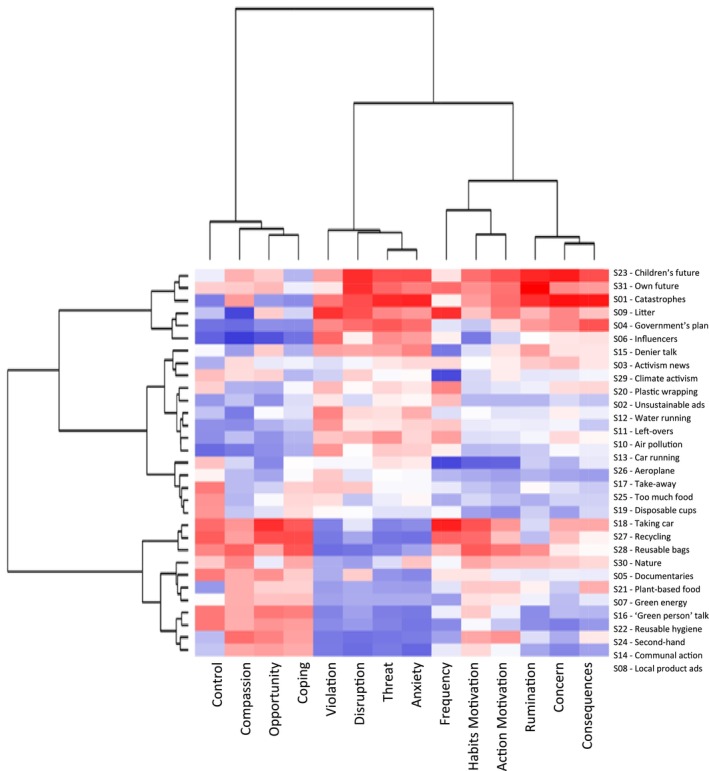
Visualisation of the standardised mean judgement for each of the 31 situations. *Note*: Each cell represents the mean score for one situation and one measure across the 303 participants. As cells become darker, they increasingly represent scores up to 2 SD smaller than the standardised mean of 0. As cells become warmer, they increasingly represent scores up to 2 SD greater than the standardised mean.

The SAM^2^ CAM not only established large differences between individuals but also between situations. Consider Figure 2, where each row shows the mean value of the 14 measures for a specific situation. On the left, situations are clustered by their similarity across the measures. At the top, measures are clustered by their similarity across the situations. Each measure varied considerably, depending on the situation.

#### Exploratory observations

As can be seen, diverse clusters of situations emerged. Whereas the cluster at the top reflects situations associated with the highest levels of climate anxiety, the clusters in the middle and at the bottom are associated with relatively moderate and low levels, respectively. Within each cluster, every situation exhibits a unique profile across the 14 measures.

Interestingly, Figure 2 also suggests that low anxiety situations are generally associated with lower concern, violation, threat and disruption, but with higher levels of opportunity, habit motivation, action motivation, control, coping and compassion. In contrast, high anxiety situations are associated with higher levels of concern, violation, threat and disruption, but with lower levels of opportunity, habit motivation, action motivation, control, coping and compassion. Rumination showed relatively mixed results. See SM‐3 for further discussion of the situation profiles.

#### Situational variability across individuals for climate anxiety

**FIGURE 3:**
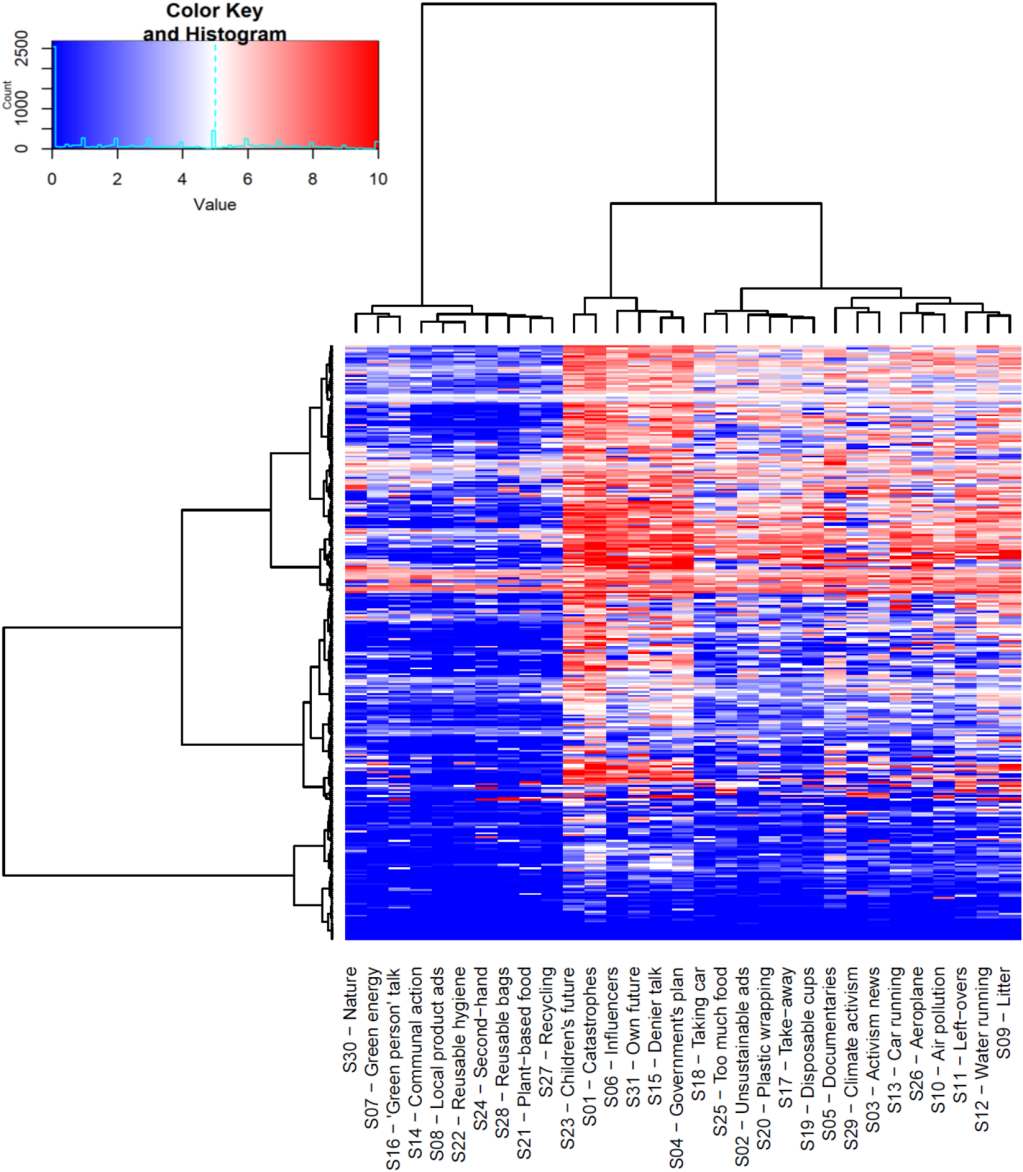
Visualisation of climate anxiety scores for each individual across the 31 situations. *Note*: Each row contains the 31 climate anxiety judgements across the 31 situations for one of the 303 participants. Table 1 presents the description for each situation (corresponding to the situation numbers shown here). As cells become redder, they represent higher climate anxiety judgements. As cells become bluer, they represent lower anxiety judgements. The hierarchical clustering dendrogram on the left groups participants according to how similarly they judged the 31 situations across situations. The hierarchical clustering dendrogram at the top groups the situations according to how similar their individual profiles are across participants.

As Figure 2 just illustrated, the 31 situations in the SAM^2^ CAM differed considerably, exhibiting very different profiles across climate anxiety and the 13 predictors. These differences suggest that climate anxiety should not be static for an individual but should vary considerably across situations. Figure 3 shows that it clearly does, where each row presents an individual's judgments for the 31 situations across the columns.

As can be seen, the 31 situations exhibited large differences in their overall levels of climate anxiety across the 303 participants, with situations on the left exhibiting low levels, situations to the right exhibiting the highest levels and situations further to the right falling in between. As predicted, most participants experienced the full range of climate anxiety responses across situations, from very low (dark blue) to very high (dark red), documenting the considerable variability of climate anxiety within an individual. Rather than climate anxiety being a static trait within an individual, it expresses itself dynamically across situations. For further discussion of situational differences, see SM‐4.

### Situation by individual interaction for climate anxiety (H2b)

As we just saw, climate anxiety exhibited large differences across situations. These effects, however, were not constant across the 303 individuals but interacted with individuals considerably. Figure 3 visualises this interaction as the different patterning of rows (and clusters of rows) across the 31 situations. Whereas the topmost cluster of individuals reported high climate anxiety for some situations, moderate anxiety for others and low anxiety for still others, the bottommost cluster reported low anxiety in most situations. In between these two clusters, other patterns of situational experience occurred, typically with high anxiety reported for a few situations and low to moderate levels reported for most others. Clearly, individuals experienced climate anxiety previously in quite different ways across the same 31 situations.

To quantify the magnitude of the situation by individual interaction for climate anxiety, we used intraclass correlations, specifically, the ICC2 random‐effects form that generalises ICC2 values to the larger population (Shrout & Fleiss, 1979). Intuitively, the ICC2 captures the average correlation across all possible pairs of participants in their judgments for a measure, thereby quantifying the situation by individual interaction. As Table 2 illustrates, the ICC2 for climate anxiety was only .21, indicating relatively low agreement in how individuals reported climate anxiety across the same situations.

As Table 2 further illustrates, large interactions occurred for most other measures as well, with ICC2 values ranging from .05 to .45. For some of these measures, agreement was very low, including action motivation, disruption, rumination, habits motivation, concern, coping, opportunity and consequences (ICC2s from .05 to .21). For other measures, agreement was somewhat higher but still relatively low, including threat, violation, frequency, compassion and control (ICC2s from .28 to .45). These values further demonstrate considerable individual differences in climate anxiety across situations, as predicted, and similarly in related SAM^2^ CAM measures.

### Construct validity (H3a)

To assess the SAM^2^ CAM's construct validity, we computed a prediction profile for each participant that established how climate anxiety was related to the 13 predictors. If the SAM^2^ measure of climate anxiety exhibits construct validity, it should exhibit relations with predictors previously observed in the literature. Specifically, climate anxiety should correlate positively with violation, threat, consequences, disruption, rumination, frequency, habits motivation, action motivation and opportunity, but should correlate negatively with control, coping, concern and compassion. As long as a majority of predictors correlate in the direction hypothesised, construct validity is established. To compute each participant's prediction profile, we correlated their judgments for climate anxiety with one predictor at a time across the 31 situations, with the resulting vector of 13 correlations constituting an individual's prediction profile.

**FIGURE 4:**
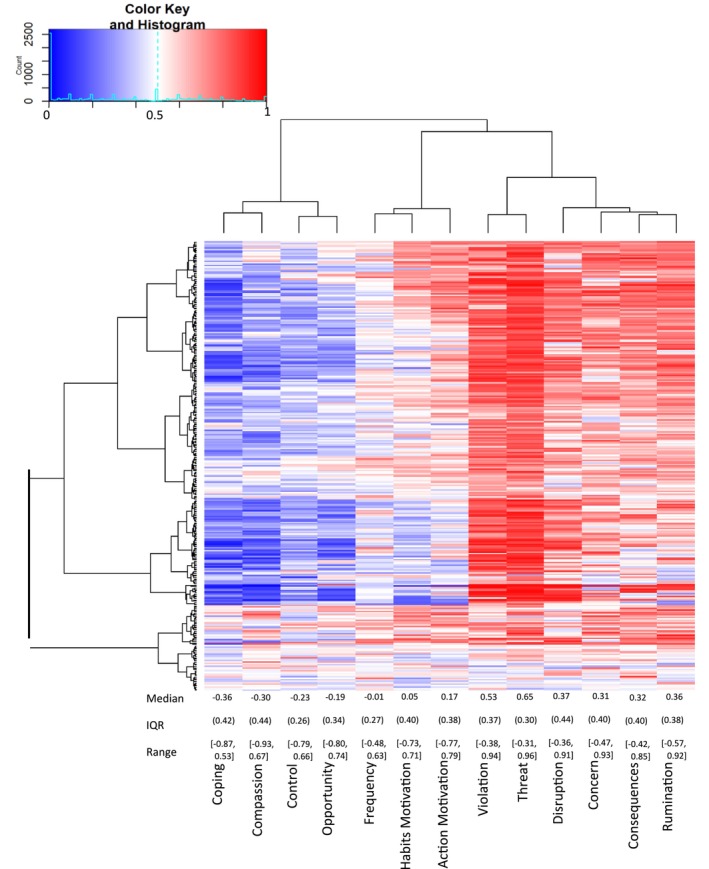
Visualisation of the 13 predictive correlations for each participant, with the median, IQR and range presented for each predictor towards the bottom. *Note*: As cells become redder, they represent correlations that are increasingly positive. As cells become bluer, they represent correlations that are increasingly negative. The hierarchical clustering dendrogram on the left groups participants according to the similarity of their predictive correlations. The hierarchical clustering dendrogram at the top groups the predictors according to how similar their individual profiles are across participants. Median for each measure shown, with IQR in round brackets, and range in square brackets.

Figure 4 displays the results of this analysis. Each row presents the prediction profile for 1 of the 303 participants, and each column presents the correlations for 1 of the 13 predictors. Each cell represents the magnitude of a correlation (red for high positive correlations, blue for high negative correlations, white for no correlation). As the median values in Figure 4 illustrate, our predictions were largely supported. Threat (*r* = .65), expectation violation (*r* = .53,), disruption (*r* = .37), rumination (*r* = .36,), consequences (*r* = .32) and action motivation (*r* = .17) all exhibited positive relations with climate anxiety across participants (all values significantly greater than 0 on Wilcoxon signed‐rank tests, one‐tailed, all *p* < .001). Conversely, coping (*r* = −.36), compassion (*r* = −.30,) and control (*r* = −.23) all exhibited negative relations (all values significantly less than 0, on Wilcoxon signed‐rank tests, one‐tailed, all *p* < .001). Unexpectedly, opportunity (*r* = −.19, *p* < .001) and concern (*r* = .32, *p* < .001), related with climate anxiety inversely to our expectations and both frequency (*r* = −.01, *p* = .17) and habits motivation (*r* = .06, *p* = .99) exhibited no clear relation to climate anxiety but instead showed large individual differences. Overall, however, these results establish construct validity for most of the SAM^2^ CAM predictors as predicted.

Importantly, large individual differences were clearly present in the prediction profiles, as captured by the interquartile ranges and ranges at the bottom of Figure 4. For every predictor, some individuals exhibited the opposite pattern from the majority. Even for the strongest predictor, threat, not all individuals exhibited positive correlations. Thus, the SAM^2^ CAM captured large individual differences in prediction profiles (illustrated by the clustering solution on the left), along with general trends (illustrated by the medians along the bottom). SM‐5 provides the correlation matrix of all raw correlations between the 14 SAM^2^ CAM measures at the group level.

### Content validity (H3b)

To assess how comprehensively the 13 predictors explained variation in climate anxiety (i.e., content validity; Cronbach, 1971), a regression analysis was performed for each participant that regressed their climate anxiety judgements for the 31 situations onto their 31 judgements for each of the 13 predictors. Variance explained (*R* ^*2*^) was computed for each individual regression and used as a measure of content validity. As outlined in Hypothesis 3b, any *R* ^*2*^ > .60 would be regarded as establishing content validity. Across the 303 individual regressions, the median *R* ^2^ was .75 (significantly greater than 0 on a Wilcoxon signed‐rank test, one‐tailed, *p* < .001, 95% CI [.73, .77]). This high level of explanation at the individual level indicates that the low ICC2 value in Table 2 for climate anxiety did not reflect random noise but instead reflected systematic individual differences (Dutriaux et al., 2023). Importantly, this high level of explained variance demonstrates that the SAM^2^ CAM exhibits high content validity, with its predictors providing comprehensive coverage of the climate anxiety construct.

### Climate anxiety and the DASS‐21 (E1)

To explore whether climate anxiety is linked to general anxiety, stress and depression, we computed correlations between the SAM^2^ CAM climate anxiety measure and each DASS‐21 subscale scores (Lovibond & Lovibond, 1995). Overall, participants reported low levels of general anxiety (*M* = 1.40, *SD* = .44), depression (*M* = 1.74, *SD* = .67) and stress (*M* = 1.82, *SD* = .61). A significant moderate correlation emerged between SAM^2^ CAM climate anxiety and the DASS‐21 general anxiety subscale (*r* = .26, *p* < .001, 95% CI [.15, .36]; see Funder & Ozer, 2019, for justification viewing .26 as a moderate effect). Similar results emerged for the DASS‐21 stress subscale, where the correlation with climate anxiety was small to medium (*r* = .20, *p* < .001, 95% CI [.09, .30]; again, see Funder & Ozer, 2019). The correlation between the DASS‐21 depression subscale and climate anxiety was small, with *r* = .15 (*p* < .001, 95% CI [.04, .26]).

## DISCUSSION

### Summary

The SAM^2^ CAM measure of climate anxiety exhibited large individual differences, with excellent test reliability of .96. Although the overall level of climate anxiety across situations was relatively low (median of 3.60 on 0‐to‐10‐point scale), individuals exhibited considerable variability in how intensely they experienced it (a range of 7.30).

The SAM^2^ CAM not only established large individual differences in climate anxiety, it also established large situational differences. Whereas some situations tended to exhibit high climate anxiety across participants, others tended to be low, with the mean climate anxiety reported for a situation ranging from 1.18 to 6.29 across situations (see SM‐2). The SAM^2^ CAM also established a rich profile of each situation's features across the 13 predictors that provides insight into its unique character. Within participants, climate anxiety tended to range from very low to very high, demonstrating the importance of situations in producing climate anxiety. Rather than being a fixed trait, climate anxiety is highly dynamic, reflecting the current situation. Finally, the SAM^2^ CAM established a large individual by situation interaction for climate anxiety, as the relatively low agreement in climate anxiety for different individuals across situations indicates. Participants varied considerably in how a specific situation contributed to their climate anxiety.

The SAM^2^ CAM exhibited good construct validity. Climate anxiety correlated positively with most predictors expected to correlate positively (e.g., threat, expectation violation, disruption). Climate anxiety similarly correlated negatively with most predictors expected to correlate negatively (e.g., coping, compassion, control). The SAM^2^ CAM also exhibited high content validity, with individual regressions finding that the 13 predictors comprehensively explained a median 75% of the variance in an individual's climate anxiety.

As we anticipated, climate anxiety as measured with the SAM^2^ CAM correlated positively but only modestly with general anxiety, as measured by the DASS‐21. Although a positive correlation was also found between SAM^2^ CAM anxiety and depression on the DASS‐21, this correlation was even smaller, suggesting that climate anxiety differs from general anxiety disorders, which typically correlate highly and frequently with depression (e.g., de Graaf et al., 2003). As suggested earlier, weak correlations between the DASS‐21 measures and the SAM^2^ CAM likely reflected shared variance at the individual trait level but divergence at the situational level. Because the SAM^2^ CAM captures variance related to a specific set of situations, it is likely to diverge significantly from measures that aren't situated, resulting in weak correlations between them. Additionally, the modest correlation between climate anxiety and general anxiety also likely reflects important differences in these two types of anxiety, as discussed earlier.

### Theoretical implications

#### Understanding climate anxiety as a situated construct

The SAM^2^ CAM demonstrates that an individual's previous experiences of climate anxiety reflect both individual and situational factors, together with their interaction. It is therefore not the case that an individual experiences similar levels of climate anxiety across different situations, or that different individuals experience the same level of climate anxiety in the same situation. Instead, different situations induce different levels of anxiety across individuals, statistically speaking, while interacting with specific individuals in different ways.

For these reasons, much important information is lost when climate anxiety is only assessed at the individual level (e.g., Clayton & Karazsia, 2020; Hogg et al., 2021). Taking situational effects into account has significant implications for how climate anxiety is understood, assessed and addressed. Future research could adapt the SAM^2^ CAM to different contexts by sampling situations relevant to different cultures, more vulnerable countries or specific demographic groups.

#### Understanding climate anxiety as a constructed emotion

Earlier we proposed that climate anxiety is a constructed emotion that emerges with increasing frequency as the result of climate change, reflecting an individual's culture, language, situational experience and biological phenotype. As these factors vary, climate anxiety takes different forms in different individuals. Individuals, for example, who have experienced climate disasters develop different forms of climate anxiety than individuals who anticipate such disasters and experience them vicariously.

From this perspective, psychometrically assessing climate anxiety should benefit from taking a situated approach. By measuring an individual's climate anxiety for specific situations where it may typically occur, along with the situational factors that predict it, the SAM^2^ CAM captures and represents the unique form that climate anxiety takes in each individual. In this manner, the SAM^2^ CAM establishes the specific way an individual's climate anxiety varies across situations, along with a unique prediction profile of the situational factors associated with it. Together, these two sources of information characterise the emotion construct that underlies different individuals' climate anxiety.

Theoretically, these findings are consistent with the view that climate anxiety is a constructed emotion that varies widely between individuals and across situations. Our findings also provide empirical evidence for Pihkala's (2020) notion that climate anxiety (like other emotions) depends on and varies between situations and cannot be explained fully by trait anxiety. Findings from the SAM^2^ CAM predictors additionally provide empirical support for the involvement of uncontrollability and unpredictability (expectation violation) in climate anxiety, as has been previously proposed (Pihkala, 2020). By adopting a constructivist view, our approach takes into account a person's physical and social context in their experiences of climate anxiety (here, *vicarious experiences* of climate change in the UK), varying widely as a function of their specific situational experience.

### Practical applications

In the UK (and similar countries), the SAM^2^ CAM could be used as a screening device for the following two purposes: (1) To identify individuals high in climate anxiety across a set of relevant situations, (2) To identify the specific situations most likely to trigger a particular individual's climate anxiety. The SAM^2^ CAM could also be used to identify the specific factors most likely associated with an individual's climate anxiety, such as threat, expectation violation or low coping ability. Once the most important situations and factors have been established for an individual, they could be used to select the coping strategies most effective for decreasing their unique form of climate anxiety. Adopting this kind of individualised approach is likely to be effective, given that it has been found useful for a wide variety of other mental health issues (e.g., Harvey et al., 2020; Hillebrand et al., 2023). This approach provides a unique opportunity for individualised support in difficult situations, without the need for pathologising a person's experiences. This could allow an individual to seek out their unique low‐anxiety situations (e.g., spending time in nature or joining climate action) or to prepare constructive coping strategies for high‐anxiety situations (e.g., preparing debrief conversations with a friend/partner when watching climate change news). This should allow individuals to cope effectively with their climate anxiety, thus preventing overwhelm and burnout‐related inaction. Further, the SAM^2^ CAM supports identifying types of situations that are typically associated with high climate anxiety across individuals, which in turn allows for community support and resilience training.

At a more general level, results from the SAM^2^ CAM could be used to inform public policy on mitigating and preventing paralysing levels of climate anxiety. Because we found that threat is strongly related to climate anxiety, for example, disaster‐focused news could be presented in alternative formats that report on those disasters but also highlight realistic action steps based on it. Similarly, because having good coping strategies is negatively related to climate anxiety, providing individuals with effective strategies is likely to reduce it. Again, these implications are more applicable to privileged populations than to vulnerable populations, given that our study assessed climate anxiety in UK adults.

### Limitations

We cannot draw causal conclusions from our correlational results. Although we were able to establish associations between climate anxiety and its predictors, as well as between climate anxiety, general anxiety, stress and depression, we cannot conclude anything about the direction of these relationships, nor make any assumptions about causation.

Similarly, our effect sizes may be somewhat inflated from recruiting participants through volunteer sampling, which could limit generalisability, as participants may have pre‐existing interests in climate change. In contrast, our sample had slightly higher education levels compared to the general UK, which may have led to reporting lower levels of climate anxiety, as lower education seems typically associated with higher climate anxiety (e.g., Poortinga et al., 2019). Because the remaining demographic data of our sample are so similar to the UK demographics in general (see Table 3), however, we are confident that our results at least approximate the climate anxiety experiences of the adult UK population. Further, due to the size of our sample, our correlational results should be generalisable from a statistical perspective (Schönbrodt & Perugini, 2013).

Additionally, given the current sample, our findings may not generalise to populations more vulnerable to climate change. The SAM^2^ CAM could, however, be easily adapted to these other populations where individuals experience personal effects of climate change more often, such as within coastal regions of the UK, countries in the global south and vulnerable island nations (see e.g., Pihkala, 2022).

Finally, although the SAM^2^ CAM provides a detailed assessment of people's recollection of climate anxiety for specific situations experienced previously, it does not assess climate anxiety in the moment. One possibility would be to assess climate anxiety using ecological momentary assessment or daily prompts (see Contreras et al., 2024). A well‐known problem with this approach, however, is that it fails to control for the types of situations where momentary assessments are captured (Dutriaux et al., 2023). An interesting possibility is to combine a SAM^2^ instrument with ecological momentary assessment, such that situations are assessed at both the type (situation) and token (momentary) levels. Another interesting question is whether one of these levels predicts climate anxiety better than the other or whether both contribute.

#### Implementing briefer versions of the SAM^2^ CAM

A final limitation of the SAM^2^ CAM is that it typically took participants about 50 minutes to complete. Although the depth and breadth of this instrument may be desirable for some applications, they may be unnecessary for others. Dutriaux et al. (2023) suggested that one way to construct a briefer version of a SAM^2^ instrument is to only collect data for the dependent variable, in this case, climate anxiety, and not for the predictors. Because we know that the SAM^2^ CAM measure for climate anxiety exhibits both construct and content validity, it is not necessary to reassess the 13 predictors, unless the predictive profiles of specific individuals are of interest. Only evaluating the 31 situations for climate anxiety should take about 4 minutes, providing a brief but reliable and valid climate anxiety instrument.

Dutriaux et al. (2023) suggested that a second way to construct a briefer SAM^2^ instrument is to reduce the number of situations and/or predictors through factor analysis. To explore this possibility, we performed two‐factor analyses on the SAM^2^ CAM. A first factor analysis of the situations revealed an optimal five‐factor solution, reducing the original instrument from 31 to 5 situations. A second factor analysis of the SAM^2^ CAM predictors similarly resulted in an optimal five‐factor solution, reducing the number of predictors from 13 to 5. When assessed on the current data set using only data for the most five representative situations and the five most representative predictors, the reduced instrument exhibited acceptable test reliability of .79. It also exhibited high construct and content validity, with the five predictors explaining a large amount of anxiety variance across situations in individual regressions (median *R* ^2^ = .60, significantly greater than 0 on a Wilcoxon signed‐rank test, one‐tailed, *p* < .001, 95% CI [.56, .64]). Details of these analyses can be found in SM‐6. With a total of 25 judgments to perform, only about 3 minutes are required to assess an individual's climate anxiety for the 5 measures across the 5 situations.

### Conclusion

Although climate anxiety was generally low in our sample, its overall level varied considerably across individuals and situations. The SAM^2^ CAM is the first instrument to evaluate situational factors in an individual's recollection of climate anxiety experienced previously in relevant situations. Findings established with the SAM^2^ CAM demonstrate the usefulness of taking a situated approach to assessing it, having implications for how climate anxiety is measured and understood.

## CONFLICT OF INTEREST STATEMENT

None of the authors have a conflict of interest to disclose.

## ETHICS STATEMENT

Ethical approval was obtained from the Ethics Committee in the College of Science and Engineering at the University of Glasgow (application number: 300210102). Participants were informed about their rights in study participation and provided informed consent.

## Supporting information


**Data S1.** Supporting information.
**SM‐1:** Introductory text presented to participants before rating the 31 situations for each measure (materials participant instructions).
**SM‐2:** The raw situation means for the 14 SAM^2^ CAM measures.
**SM‐3:** A guide to establishing and interpreting the profiles for specific situations from the SAM^2^ CAM.
**SM‐4:** Detailed discussion of situation effects on climate anxiety.
**SM‐5:** Correlation matrix for all SAM^2^ CAM raw data, taken across participants.
**SM‐6:** Analyses and recommendations for a shorter SAM2 CAM instrument.
**SM‐7:** Overview and schematic of the SAM^2^ CAM situation selection and generation process.

## Data Availability

The data that support the findings of this study are openly available in Open Science Framework at https://osf.io/2zg5u/overview?view_only=e5f93c90da4a4c31b17ac189e7a2c642.
