# Supplementary material for: Developing and evaluating a situated psychometric instrument for assessing climate anxiety: The SAM2 CAM
Source: Appl Psychol Health Well Being. 2026 Feb 4;18(1):e70125. doi: 10.1111/aphw.70125 (PMC12872338; doi:10.1111/aphw.70125)
Supplement: Supplementary file 1 — Data S1. Supporting information. SM‐1: Introductory text presented to participants before rating the 31 situations for each measure (materials participant instructions). SM‐2: The raw situation means for the 14 SAM2 CAM measures. SM‐3: A guide to establishing and interpreting the profiles for specific situations from the SAM2 CAM. SM‐4: Detailed discussion of situation effects on climate anxiety. SM‐5: Correlation matrix for all SAM2 CAM raw data, taken across participants. SM‐6: Analyses and recommendations for a shorter SAM2 CAM instrument. SM‐7: Overview and schematic of the SAM2 CAM situation selection and generation process. [file APHW-18-0-s001.pdf]

SM-1
Introductory text presented to participants before rating the 31 situations for each measure.

Measure	Introductory Text
**Anxiety**	"In a moment, you will receive a series of 31 situations. For each situation, you will be asked to rate: **When this situation occurs, how much anxiety do you experience about climate change?** If you haven't previously experienced the situation, just imagine that it occurs sometime in the future, and then evaluate what you imagine. A little more detail about what we mean by **anxiety** follows next.<br><br>**Anxiety** is a negative response to anticipated future situations that are likely difficult in some way. Often, this negative response includes unpleasant bodily states, such as a fast heart rate, difficulty breathing, muscle tension, and intense arousal. Anxiety is often associated with worry, rumination, and feeling upset about a difficult future event. Situations that produce anxiety are typically not perceived as desirable but are instead perceived as anticipated difficulties one would prefer to avoid. Although some people don't currently experience anxiety about the climate change, other people increasingly do."
**Frequency**	"In a moment, you will receive a series of 31 situations. For each situation, you will be asked to rate: **How frequently do you experience this situation?** A little more detail about what we mean by **situation frequency** follows next.<br><br>**Situation frequency** refers to how often you experience a situation. Some situations you may have never experienced. Others you may experience about once a year, once a month, or once a week. Some you may experience everyday or multiple times each day."
**Concern**	"In a moment, you will receive a series of 31 situations. For each situation, you will be asked to rate: **How concerned are other people in this situation about climate change and sustainability?** If you haven't previously experienced the situation, just imagine that it occurs sometime in the future, and then evaluate what you imagine. A little more detail about what we mean by **other people's concern** follows next.<br><br>People you may know may vary widely in **how concerned they are about climate change and sustainability**. Some people may not be concerned at all, whereas others may be intensely concerned. Furthermore, the amount of concern you encounter may vary widely across the situation you're in at the moment, depending on the people present."

Violation "In a moment, you will receive a series of 31 situations. For each situation, you will be asked to rate: **How much does this situation violate your expectations?** If you haven't previously experienced the situation, just imagine that it occurs sometime in the future, and then evaluate what you imagine. A little more detail about what we mean by **expectation violation** follows next.

An **expectation violation** can potentially take numerous forms, including: (1) an unexpected or "surprise" situation (e.g., an unexpected bill in the mail for something you didn't purchase), (2) an unexpected outcome for an expected situation (e.g., an unexpectedly high charge on a monthly bill), (3) an expected violation of a desirable outcome that one would prefer to not see violated (e.g., receiving a high bill as expected that violates the desire to minimise expenditures), and (4) the violation of a social norm (e.g., a friend asks you to pay their utility bill). Please feel free to draw on any of these possibilities to assess how your assessments of expectation violation next."

Threat "In a moment, you will receive a series of 31 situations. For each situation, you will be asked to rate: **How threatened do you feel by what happens in this situation?** If you haven't previously experienced the situation, just imagine that it occurs sometime in the future, and then evaluate what you imagine. A little more detail about what we mean by **threat** follows next.

A **threat** can take numerous forms, including something that could potentially cause: (1) physical pain or injury (e.g., falling on an icy pavement), (2) damage to your property (e.g., damage to your car due to icy roads), (3) danger to your loved ones (e.g., being out in the cold for long without protection), or (4) an unpleasant mental state (e.g., fear about getting lost while hiking in a snow storm). Please feel free to draw on these kinds of threat and others in your assessments of threat next."

Opportunity "In a moment, you will receive a series of 31 situations. For each situation, you will be asked to rate: **How much of an opportunity do you see in this situation to do something constructive?** If you haven't previously experienced the situation, just imagine that it occurs sometime in the future, and then evaluate what you imagine. A little more detail about what we mean by **opportunity** follows next.

An opportunity to do something constructive refers to a situation where you can achieve something positive. Such opportunities can include connecting with others, contributing to your community, supporting family and friends, and protecting the environment. Such opportunities may also be more personal, such as experiencing personal growth, establishing a purpose, discovering meaning, and achieving personal goals. Many other potentially positive outcomes may come to mind for you as well."

**Motivation Habits**	"In a moment, you will receive a series of 31 situations. For each situation, you will be asked to rate: **How much does being in this situation motivate you to adopt sustainable habits?** If you haven't previously experienced the situation, just imagine that it occurs sometime in the future, and then evaluate what you imagine. A little more detail about what we mean by **motivation to adopt sustainable habits** follows next.<br><br>**Motivation** is a reason or feeling that makes you want to perform a behaviour. **Sustainable habits** are behaviours performed regularly to ensure that we don't permanently deplete natural resources or damage the environment supporting our existence. Sustainable habits maintain a balance between human activity and the environment, allowing both to coexist and thrive. Thus, **motivation to adopt sustainable habits** refers to motivation that makes you want to perform eco-friendly behaviours, perhaps replacing behaviours that are less environmentally friendly."
**Motivation Action**	"In a moment, you will receive a series of 31 situations. For each situation, you will be asked to rate: **How much does being in this situation motivate you to perform social action that promotes sustainability?** If you haven't previously experienced the situation, just imagine that it occurs sometime in the future, and then evaluate what you imagine. A little more detail about what we mean by **motivation to perform social action that promotes sustainability** follows next.<br><br>**Motivation** is a reason or feeling that makes you want to perform a behaviour. **Social action that promotes sustainability** refers to coming together with others and acting as a group to prevent depletion of natural resources and damage to the environment. This kind of social action may also aim to develop social awareness and influence government policy related to climate change and sustainability. Additionally, social action may take the form of volunteering, joining community organisations, joining peer networks, fundraising, protesting, and so forth. Feel free to draw on any of these possibilities to assess how much each situation that follows motivates you to engage in social action that promotes sustainability."
**Control**	"In a moment, you will receive a series of 31 situations. For each situation, you will be asked to rate: **How much control do you believe you have over what happens in this situation?** If you haven't previously experienced the situation, just imagine that it occurs sometime in the future, and then evaluate what you imagine. A little more detail about what we mean by **personal control** follows next.<br>**Personal control** refers to how much you believe that you have the ability and the means to influence a situation you're currently experiencing. You may be able to influence either what happens as the situation unfolds or what its final outcome is. You could attempt to control a situation through your personal actions, through your ability to influence the actions of others, through your ability to alter physical aspects of the environment, and so forth. Alternatively, you may believe that you have little or no ability to influence a situation in any of these ways, with other factors having control over it instead (e.g., other individuals, social institutions, environmental causes, etc.)."

Coping "In a moment, you will receive a series of 31 situations. For each situation, you will be asked to rate: **How effectively are you able to cope with this situation?** If you haven't previously experienced the situation, just imagine that it occurs sometime in the future, and then evaluate what you imagine. A little more detail about what we mean by **coping effectiveness** follows next.

Coping effectiveness refers to how effectively and confidently you handle a situation to achieve an outcome that is satisfactory. In some situations, you may know exactly what to do, have high confidence that you can resolve the situation effectively, and succeed in achieving a satisfactory outcome (high coping effectiveness). In other situations, you may have no idea what to do, have low confidence that you can resolve the situation effectively, and fail to achieve a satisfactory outcome (low coping effectiveness)."

Rumination "In a moment, you will receive a series of 31 situations. For each situation, you will be asked to rate: **How likely are you to ruminate about this situation?** If you haven't previously experienced the situation, just imagine that it occurs sometime in the future, and then evaluate what you imagine. A little more detail about what we mean by **rumination** follows next.
Rumination refers to continually thinking about a situation when it's not actually occurring. For some situations, you may ruminate about them immediately after they occur. For others, you may ruminate about them just before they occur. Sometimes rumination arises spontaneously even when a situation hasn't just occurred or isn't just about to occur."

Compassion "In a moment, you will receive a series of 31 situations. For each situation, you will be asked to rate: **How judgmental / compassionate are you about yourself and other people in this situation?** If you haven't previously experienced the situation, just imagine that it occurs sometime in the future, and then evaluate what you imagine. A little more detail about what we mean by **compassion and judgmentalness** follows next.

Compassion refers to how warm and understanding you are toward yourself and others when various situations arise, especially difficult ones. The opposite of being compassionate in these situations is being **judgmental**, negatively criticising your actions, thoughts, emotions, and who you are, along with similarly being judgmental about others. People vary widely in how compassionate versus judgmental they are toward themselves and others in particular situations."

**Consequences**	"In a moment, you will receive a series of 31 situations. For each situation, you will be asked to rate: **How much do you think about the consequences of what happens in this situation for climate change and sustainability?** If you haven't previously experienced the situation, just imagine that it occurs sometime in the future, and then evaluate what you imagine. A little more detail about what we mean by **consequences** follows next.<br><br>As many situations occur, your actions in them potentially have **consequences for climate change and sustainability** across the Earth's diverse ecologies. These **consequences** can take many forms. Sea levels are rising due to the polar caps melting. Extreme weather is causing droughts, heat waves, flooding, uncontrollable fires, and other serious outcomes. The seasons as we know them are shifting around the world. Places that were once inhabitable are becoming uninhabitable due to flooding, lack of water, unbearable heat, and so forth. Of interest next is how much you think about potential consequences for climate change and sustainability as you're acting in various situations."
**Disruption**	"In a moment, you will receive a series of 31 situations. For each situation, you will be asked to rate: **How much does this situation disrupt your life?** If you haven't previously experienced the situation, just imagine that it occurs sometime in the future, and then evaluate what you imagine. A little more detail about what we mean by **disruption** follows next.<br>**Disruption** refers to how much a situation interferes with what you are currently doing or what you had planned. It also refers to how upset you become by not being able to do these other things and instead having to deal with the disruption. Some events may be extremely disruptive to your day-to-day life, whereas others may not interfere with anything at all."

SM-2

The raw situation means for the 14 SAM² CAM measures.

	Anxiety	Freq.	Concern	Violation	Threat	Opp.	Motiv_ Habits	Motiv_ Action	Control	Coping	Rumin.	Comp.	Conseq.	Disruption
**S01 Catastrophes**	6.29	4.80	6.62	5.64	6.20	3.29	6.71	5.99	2.25	4.77	6.39	1.95	7.66	3.76
**S02 Unsustain. ads**	3.36	5.68	3.85	3.89	3.15	3.17	4.66	3.94	2.88	5.88	3.23	-.83	4.71	1.95
**S03 Activism news**	3.79	5.22	4.90	2.97	3.28	4.54	5.52	4.80	3.40	6.17	4.64	.85	5.56	2.20
**S04 Gov.'s plan**	5.26	4.06	5.43	5.37	5.48	3.13	4.86	4.96	1.98	4.76	5.18	-2.34	7.03	3.50
**S05 Documentaries**	4.09	4.42	4.77	2.18	2.37	4.55	6.57	5.25	6.63	7.54	4.76	2.31	5.69	1.47
**S06 Influencers**	4.60	4.80	4.48	5.93	4.66	2.22	3.94	4.23	1.58	4.42	4.04	-3.15	5.61	2.42
**S07 Green energy**	1.92	4.07	3.71	1.93	1.42	5.57	6.18	5.14	2.88	6.97	4.20	1.63	6.13	1.57
**S08 Local prod. ads**	1.18	4.18	3.02	1.41	0.88	6.35	6.00	4.56	3.96	7.51	3.07	1.70	4.35	1.06
**S09 Litter**	4.67	8.09	5.43	6.73	4.75	5.66	6.25	5.85	3.86	5.75	4.87	-2.97	5.93	3.75
**S10 Air pollution**	3.85	6.16	4.61	4.41	4.58	3.20	5.14	4.37	2.64	5.33	3.95	-.90	5.38	2.87
**S11 Left-overs**	3.88	5.51	4.12	5.17	3.29	3.68	5.28	4.50	2.68	5.60	3.85	-1.77	4.75	2.45
**S12 Water running**	4.39	3.99	4.38	5.46	3.41	4.80	5.50	4.37	3.90	5.94	3.76	-2.05	5.14	2.61
**S13 Car running**	3.94	4.83	4.16	5.17	3.72	3.15	4.70	4.02	1.78	5.25	3.47	-2.07	5.11	2.27
**S14 Communal act.**	1.48	2.99	3.39	1.40	0.79	6.79	6.58	5.66	3.71	7.64	3.68	2.47	5.53	1.10
**S15 Denier talk**	4.97	2.28	4.49	4.98	4.34	5.66	5.12	4.91	5.16	5.27	5.12	-1.46	5.56	3.01

**S16 ‘Green person’**	2.11	2.91	3.56	2.01	1.59	5.81	5.91	4.86	5.41	7.21	3.89	1.62	5.06	1.58
**S17 Take-away**	3.19	3.32	3.32	4.35	2.89	3.50	4.52	3.75	5.63	6.31	3.20	-.96	4.31	2.05
**S18 Taking car**	2.85	4.43	3.26	3.57	2.45	4.40	4.41	3.59	7.87	6.81	3.51	-.76	4.88	2.26
**S19 Dispos. cups**	3.37	3.16	3.79	4.03	2.64	4.79	5.04	4.05	7.95	6.90	3.16	-1.04	4.84	1.79
**S20 Plastic wrap.**	3.61	6.61	4.57	4.60	3.55	3.60	5.04	4.40	6.49	6.25	3.88	-1.14	5.71	2.32
**S21 Plant-based**	1.62	5.00	4.06	2.19	1.25	6.57	5.84	4.47	8.50	7.05	3.79	1.63	5.10	2.70
**S22 Reusable hyg.**	1.43	4.26	3.55	1.53	.92	6.94	6.16	4.50	8.55	8.11	2.76	1.65	4.55	1.49
**S23 Children’s fut.**	5.72	5.05	6.51	4.98	5.60	5.65	7.25	6.26	4.91	5.33	6.46	1.58	7.08	4.05
**S24 Second-hand**	1.40	2.56	2.93	1.64	0.98	6.51	5.45	4.11	8.58	7.70	2.78	1.64	4.26	1.43
**S25 Too much food**	3.19	4.17	3.90	4.44	2.81	4.17	4.83	3.92	8.47	7.01	4.11	-.96	4.77	2.75
**S26 Aeroplane**	3.56	1.44	3.45	3.38	3.38	3.02	3.65	3.18	6.81	6.37	3.49	-.51	5.07	2.24
**S27 Recycling**	1.71	8.34	5.08	1.52	.99	8.06	7.57	5.61	8.87	8.64	3.62	2.04	6.17	2.07
**S28 Reusable bags**	1.32	7.20	4.87	1.38	.76	7.60	7.29	5.22	9.24	8.83	3.32	1.86	5.42	1.54
**S29 Climate act.**	3.32	1.49	4.01	2.76	2.84	5.57	5.08	4.81	6.90	5.34	3.78	.82	5.19	2.68
**S30 Nature**	2.02	5.78	4.42	1.21	1.04	6.19	7.50	5.92	8.29	8.69	5.29	2.95	5.34	1.12
**S31 Own future**	5.03	7.07	5.38	3.88	5.25	5.95	6.86	5.96	6.51	6.11	7.13	1.13	6.30	4.03

Note. Abbreviations Measures: Freq. = Frequency; Opp. = Opportunity; Motiv_Habits = Motivation Habits; Motiv_Action = Motivation Action; Rumin. = Rumination; Comp. = Compassion; Conseq. = Consequences. Abbreviations Situations: S02 Unsustain. ads = S02 Unsustainable ads; S04 Gov.’s plan = S04 Government’s plan; S08 Local prod. Ads = S08 Local product ads; S14 Communal act. = S14 Communal action; S16 ‘Green person’ = S16 ‘Green person’ talk; S19 Dispos. Cups = S19 Disposable cups; S20 Plastic wrap. = S20 Plastic wrapping; S21 Plant-based = S21 Plant-based food; S22 Reusable hyg. = S22 Reusable hygiene; S23 Children’s fut. = S23 Children’s future; S29 Climate act = S29 Climate activism.

SM-3
A guide to the profiles for specific situations

The profiles in Figure 2 offer insight into the experience of specific situations. Each situation can be interpreted for climate anxiety and each of the 13 predictors.

Consider *S01 - Hearing about climate catastrophes on the news*. As can be seen in Figure 2, not only do people experience a high level of anxiety in this situation, they also feel highly threatened, are highly concerned about its consequences, and ruminate about what is happening. Simultaneously, people are unable to cope, experience little control, and perceive little opportunity to do something constructive. For an interesting, and perhaps somewhat paradoxical contrast, consider *S31 - Thinking about my future*. Here, people again experience high levels of anxiety, concern and rumination, but now experience higher levels of coping, control, and opportunity.

One can similarly explore each remaining situation, examine its profile across measures, and gain insight into how people experience it. These profiles could also be used for developing interventions to reduce climate anxiety, tailored to the specific features present in a situation.

SM-4

Detailed discussion of situation effects on climate anxiety

In the left-most cluster of columns in Figure 3, the respective situations elicited relatively low climate anxiety judgments, including *S07 - green energy*, *S08 - local product ads*, *S14 - communal action*, *S16 - 'green person' talk*, *S21 - plant-based food*, *S22 - reusable hygiene*, *S24 - second-hand*, *S27 - recycling*, *S28 - reusable bags*, and *S30 - nature*. In contrast, the next cluster of situations to the right elicited relatively high climate anxiety judgments, including *S01 - catastrophes*, *S04 - government's plan*, *S09 - litter*, *S12 - water running*, *S15 - denier talk*, *S23 - children's future*, and *S31 - own future*. Notably, however, most of the other situations in the right half of Figure 3 exhibited mixed responses of high and low climate anxiety judgments, including *S02 - unsustainable ads*, *S03 - activism news*, *S05 - documentaries*, *S06 - influencers*, *S10 - air pollution*, *S11 - left-overs*, *S13 - car running*, *S17 - take-away*, *S18 - taking car*, *S19 - disposable cups*, *S20 - plastic wrapping*, *S25 - too much food*, *S26 - aeroplane*, and *S29 - climate activism*. These general patterns indicate that an individual's climate anxiety is not constant across situations but varies widely.

Interestingly, it appears that higher climate anxiety was often associated with situations that are threatening but may not affect an individual's life circumstances directly: *S01 - catastrophes*, *S04 - government's plan*, *S12 - water running*, *S15 - denier talk*. Situations that posed direct threats to one's circumstances were in the minority: *S23 - children's future*, *S31 - own future*. Interestingly, all the situations eliciting higher climate anxiety are generally experienced as not very controllable.

In comparison, situations associated with lower climate anxiety were mostly those with immediate effects to one's personal circumstances and over which an individual has control: *S14 - communal action*, *S16 - 'green person' talk*, *S21 - plant-based food*, *S22 - reusable hygiene*, *S24 - second-hand*, *S27 - recycling*, *S28 - reusable bags*, *S30 - nature*. Situations associated with lower climate anxiety that did not have an immediate effect on personal circumstances and that lay outside of one's control were in the minority, including *S07 - green energy*, *S08 - local product ads*.

For the situations with mixed climate anxiety scores, six have no direct effect on someone's life (*S02 - unsustainable ads*, *S03 - activism news*, *S05 - documentaries*, *S06 - influencers*, *S11 - left-overs*, *S13 - car running*), while eight situations may have a direct effect (*S10 - air pollution*, *S17 - take-away*, *S18 - taking car*, *S19 - disposable cups*, *S20 - plastic wrapping*, *S25 - too much food*, *S26 - aeroplane*, *S29 - climate activism*). Interestingly, exactly half of the mixed scored situations can be controlled by an individual (*S17 - take-away*, *S18 - taking car*, *S19 - disposable cups*, *S20 - plastic wrapping*, *S25 - too much food*, *S26 - aeroplane*, *S29 - climate activism*), while the other half can generally not (*S02 - unsustainable ads*, *S03 - activism news*, *S05 - documentaries*, *S06 - influencers*, *S10 - air pollution*, *S11 - left-overs*, S*13 - car running*). Thus, is appears that participants generally experienced higher climate anxiety in situations that were not under their control and that posed a general threat. In contrast, they seemed to experience lower climate anxiety in situations they could control to some extent and that had a direct effect on their personal lives. Later correlational analyses will partially support this observation.

SM-5

Correlation matrix for all SAM² CAM raw data, taken across participants.

	Anxiety	Frequency	Concern	Violation	Threat	Opportunity	Motivation Habits	Motivation Action	Control	Coping	Rumination	Compassion	Consequences	Disruption
**Anxiety**	1.00	-	-	-	-	-	-	-	-	-	-	-	-	-
**Frequency**	0.12	1.00	-	-	-	-	-	-	-	-	-	-	-	-
**Concern**	0.37	0.22	1.00	-	-	-	-	-	-	-	-	-	-	-
**Violation**	0.57	0.07	0.30	1.00	-	-	-	-	-	-	-	-	-	-
**Threat**	0.67	0.13	0.34	0.64	1.00	-	-	-	-	-	-	-	-	-
**Opportunity**	-0.01	0.17	0.13	-0.07	-0.05	1.00	-	-	-	-	-	-	-	-
**Motivation Habits**	0.23	0.26	0.27	0.12	0.17	0.53	1.00	-	-	-	-	-	-	-
**Motivation Action**	0.32	0.24	0.30	0.21	0.27	0.38	0.61	1.00	-	-	-	-	-	-
**Control**	-0.12	0.04	0.04	-0.16	-0.14	0.38	0.25	0.15	1.00	-	-	-	-	-
**Coping**	-0.34	0.08	-0.05	-0.33	-0.37	0.23	0.14	0.03	0.36	1.00	-	-	-	-
**Rumination**	0.44	0.21	0.33	0.33	0.46	0.22	0.41	0.47	0.06	-0.11	1.00	-	-	-
**Compassion**	-0.24	0.06	0.01	-0.44	-0.31	0.31	0.25	0.16	0.24	0.31	0.02	1.00	-	-
**Consequences**	0.42	0.21	0.36	0.31	0.38	0.26	0.49	0.48	0.05	-0.08	0.53	0.02	1.00	-
**Disruption**	0.51	0.13	0.28	0.44	0.57	0.06	0.18	0.30	-0.01	-0.28	0.40	-0.17	0.35	1.00

SM-6

Analyses and recommendations for a shorter SAM[2] CAM instrument

SM-6.1. Reducing number of Situated Action Cycle predictors

Based on our exploratory factor analysis (orthogonal – varimax rotation), the 5-factor solution offers the best conceptual fit (see image below).

```
[illegible]
> print(df_fa_5$loadings, cutoff = .3)

Loadings:
              MR4    MR1    MR2    MR3    MR5   
Frequency                                  0.356
Concern                                    0.473
Violation            0.343  0.746               
Threat               0.623  0.470               
Opportunity   0.536                0.318        
Motiv_Habits  0.833                             
Motiv_Action  0.625                             
Control                            0.832        
Coping              -0.378         0.355        
Rumination    0.399  0.493                0.308
Compassion                 -0.565               
Consequences  0.474  0.372                0.331
Disruption           0.611                      

                 MR4   MR1   MR2   MR3   MR5
SS loadings    1.969 1.557 1.329 0.988 0.839
Proportion Var 0.151 0.120 0.102 0.076 0.065
Cumulative Var 0.151 0.271 0.374 0.449 0.514
```

For a conservative 5-predictor measure directly informed by the 5-factor solution, we propose the following predictors (informed by factor loadings):

- Motivation Habits (MR4);
- Threat (MR1);
- Violation (MR2);
- Control (MR3);
- Concern (MR5).

To test whether the reduced set of predictors still manage to explain a preferrable amount of climate anxiety variance, we conducted a simple linear regression. Here, Anxiety was the dependent variable, with the five predictors retained from the exploratory factor analysis as predictors, with no random intercepts or interactions. The five Situated Action Cycle predictors retained after the exploratory factor analysis together explain about a median 60% of anxiety variance, which is less than the original 13 factors (which explained 75%). For a detailed analysis, see R script "@@6-SAM2_climate_anxiety-factor_analysis.R" and "@@5-SAM2_climate_anxiety-individual_regressions_v3.R" (line 171).

For a more comprehensive measure, including some more conceptually important predictors, we recommend the following seven predictors (informed by both, factor loadings and theoretical importance for climate anxiety):

- Threat;
- Disruption;
- Violation;
- Control;
- Motivation Habits;
- Opportunity;

- Frequency.

Again, we conducted a simple linear regression to assess the variance explained by the seven suggested predictors. Here, Anxiety was the dependent variable, with the seven suggested predictors from the exploratory factor analysis as predictors, with no random intercepts or interactions. The seven suggested Situated Action Cycle predictors together explain about 69% of anxiety variance, which is less than the original 13 factors (which explained 75%) but more than the conservative five predictor solution above. For a detailed analysis, see R script "@@6-SAM2_climate_anxiety-factor_analysis.R".

SM-6.2. Reducing number of SAM² CAM situations

Based on our exploratory factor analysis (orthogonal – varimax rotation), the 5-factor solution offers the best conceptual fit (see image below).

```
> print(df_fa_sit_5$loadings, cutoff = .3)

Loadings:
    MR2    MR1    MR4    MR5    MR3
s01         0.652         0.407
s02         0.704
s03  0.494  0.492
s04         0.756
s05  0.623
s06         0.776
s07  0.708  0.404
s08  0.776
s09         0.725
s10         0.743
s11         0.765
s12         0.678  0.376
s13         0.806
s14  0.800
s15         0.481  0.304         0.376
s16  0.714                       0.334
s17         0.529  0.577
s18  0.349  0.362  0.655
s19  0.358  0.361  0.678
s20         0.588  0.512
s21  0.666         0.359
s22  0.784         0.425
s23         0.481         0.574
s24  0.707         0.436
s25  0.332  0.383  0.652
s26         0.342  0.566
s27  0.784
s28  0.797         0.360
s29  0.480                       0.484
s30  0.778
s31  0.346  0.386         0.581

                 MR2   MR1   MR4   MR5   MR3
SS loadings    7.563 7.325 3.765 1.302 1.049
Proportion Var 0.244 0.236 0.121 0.042 0.034
Cumulative Var 0.244 0.480 0.602 0.644 0.678
```

For a conservative 5-situation measure directly informed by the 5-factor solution, we propose the following situations (informed by factor loadings):

- S14 – Communal action (MR2);
- S13 – Car running (MR1);
- S19 – Disposable cups (MR4);
- S31 – Own future (MR5);
- S29 – Climate activism (MR3).

For a more comprehensive measure, including some more conceptually important situations, we recommend the following seven situations (informed by both, factor loadings and theoretical importance for climate anxiety):

- S14 – Communal action;
- S13 – Car running;
- S19 – Disposable cups;
- S31 – Own future;
- S29 – Climate activism;
- S01 – Catastrophes;
- S23 – Children's future.

Finally, the existing SAM² CAM situations could be revised and combined to create a shorter, but all-round informative measure, as informed by the literature on climate anxiety. Thus, we suggest the retention of some, revision/ combining of other situations, as follows:

- S01 – Catastrophes;
- S31 – Own future;
- S23 – Children's future;
- S04 – Government's plan;
- NEW combination of situations: "Witnessing unsustainable behaviour (e.g., seeing someone leave their car running while parked).";
- S14 – Communal action;
- S30 – Nature;
- NEW combination of situations: "Engaging in less common sustainable behaviours (e.g., buying clothes second hand).";
- S19 – Climate activism (MR3);
- NEW combination of situations: "Engaging in common sustainable behaviours (e.g., taking the car when I have a choice not to).";

To use a shorter version of the SAM² CAM, we propose that, depending on the purpose and goal of its use, the following options:

1) Use **all** original SAM² CAM situations, but **no** Situated Action Cycle predictors (predicted assessment length: **± 4 minutes**).
    - This will be of use when only the intensity of climate anxiety is of interest, with
    - no interest in the predictors of climate anxiety, but
    - a comprehensive assessment of climate anxiety is desired.

2) Use one of the **reduced** sets of situations (choose depending on desired complexity of climate anxiety to be assessed), together with **reduced** set of Situated Action Cycle predictors (predicted assessment length: ± **8 minutes**).
    - This will be of use when intensity of climate anxiety is of interest,
    - as well as a sense of the most important predictors of climate anxiety, but
    - a less comprehensive assessment of climate anxiety is sufficient.

3) Use one of the **reduced** sets of situations (choose depending on desired complexity of climate anxiety to be assessed), but **no** Situated Action Cycle predictors (predicted assessment length: **± 1.5 minutes**).
    - This will be of use when only intensity of climate anxiety is of interest, with

- no interest in the predictors of climate anxiety, and
- a less comprehensive assessment of climate anxiety is sufficient.

Table SM-6.3. Suggested judgements from exploratory factor analysis grouped by Situated Action Cycle (SAC) phases, with corresponding intraclass correlations.

SAC Phase	Judgement Name/ Query/ Values/ Labels	ICC2	ICC3	ICC3k
Affect	**Anxiety**<br>When this situation occurs, how much anxiety do you experience about climate change?<br>(0 to 10)/ (No anxiety at all, Moderate anxiety, Extreme anxiety)	.24	.47	.86
Environment	**Frequency**<br>How frequently do you experience each of the following situations?<br>(0 to 10)/ (Never, Once a month, Multiple times a day)	.31	.36	.79
Self-relevance	**Opportunity**<br>How much of an opportunity do you see in this situation to do something constructive?<br>(0 to 10)/ (No opportunity at all, Moderate opportunity, Extremely good opportunity)	.40	.21	.65
Environment	**Concern**<br>How concerned are other people in this situation about climate change and sustainability?<br>(0 to 10)/ (Not concerned at all, Moderately concerned, Extremely concerned)	.17	.29	.74
Self-relevance	**Violation**<br>How much does this situation violate your expectations?"<br>(0 to 10)/ (No violation at all, Moderate violation, Extreme violation)	.21	.36	.80
Self-relevance	**Threat**<br>How threatened do you feel by what happens in this situation?"<br>(0 to 10)/ (Not threatened at all, Moderately threatened, Extremely threatened)	.11	.44	.85
Affect	**Motivation Habits**<br>How much does being in this situation motivate you to adopt sustainable habits?<br>(0 to 10)/ (Not at all, Moderately, Extremely)	.15	.40	.83
Action	**Control**<br>How much control do you believe you have over what happens in this situation?<br>(0 to 10)/ (No control at all, Moderate control, Full control)	.41	.19	.62
Outcome	**Disruption**<br>How much does this situation disrupt your life?<br>(0 to 10)/ (Not at all, Moderately, Extremely)	.22	.26	.71

Note. The left column shows the phase of the Situated Action Cycle from which each measure was sampled. The second column presents the climate anxiety item used in the SAM^2 CAM, followed by the suggested items for the SAM^2 predictors identified through exploratory factor analysis (each preceded by its name in bold). Below each assessment item are the end points of the continuous slider scaled used to assess it, together with the scale's labels. The ICC2 is the interrater agreement for each measure across the seven suggested situations retained from exploratory factor analysis, treating participants as random effects. The ICC3 is the coherence of the seven suggested situations as test items on the SAM^2 CAM (i.e., how consistently they order participants), with the situations treated as fixed effects. The ICC3k is Cronbach's alpha for the overall score of each measure (aggregated across the seven suggested situations for each participant), capturing the measure's test reliability in ordering participants. Note, that Disruption may not be a relevant predictor in areas and individuals less affected by climate change but may be important for areas/ individuals who have been exposed (e.g., those living in the Global South). Also note that the ICC3k for Anxiety is good, however, other measures with ICC3k below .80 may not be acceptable. We suggest that Spearman-Brown formula (e.g., see Warrens, 2017) be used to identify the number of situations required for an ICC3k of .80 or above, if the affected predictors are to be used.

SM-7

Schematic of the Situation Selection Process for the SAM^2 CAM

Literature search on climate anxiety and the types of experiences and situations linked to it.

↓

Identified four domains commonly mentioned to be linked to climate anxiety: *Media exposure/ exposure to climate change information*; *experiencing climate change consequences or environmental pollution*; *social interactions about climate change*; and *sustainable behaviours and climate action (negatively linked).*

↓

First collection of potential situation types, based on the four domains. Including situation types for each domain likely to induce or reduce climate anxiety.

Revised initial situation types to more concrete situations, combining ones likely to induce and reduce climate anxiety. Overlapping types of situations were removed.

Further revised situations to reduce overlap and remove less relevant/ salient situations.

Discussions with colleagues with lived experience of climate anxiety to refine a final set of situations.

Situation Selection Process for the SAM² CAM

0. Literature search: identified four domains linked to climate anxiety (Sep – Nov 2021)

Domain 1: Media exposure/ exposure to climate change information

Luo, Y., & Zhao, J. (2021). Attentional and perceptual biases of climate change. *Current Opinion in Behavioral Sciences, 42*, 22-26. https://doi.org/10.1016/j.cobeha.2021.02.010

Maran, D. A., & Begotti, T. (2021). Media exposure to climate change, anxiety, and efficacy beliefs in a sample of Italian university students. *International Journal of Environmental Research and Public Health, 18*(17), 1-11. https://doi.org/10.3390/ijerph18179358

Ogunbode, C. A., Doran, R., & Böhm, G. (2020). Exposure to the IPCC special report on 1.5 °C global warming is linked to perceived threat and increased concern about climate change. *Climatic Change, 158*(3-4), 361-375. https://doi.org/10.1007/s10584-019-02609-0

Usher, K., Durkin, J., & Bhullar, N. (2019). Eco-anxiety: How thinking about climate change-related environmental decline is affecting our mental health. *International Journal of Mental Health Nursing, 28*(6), 1233-1234. https://doi.org/10.1111/inm.12673

Domain 2: Experiencing climate change consequences or environmental pollution

Dodd, W., Scott, P., Howard, C., Scott, C., Rose, C., Cunsolo, A., & Orbinski, J. (2018). Lived experience of a record wildfire season in the northwest territories, Canada. *Canadian Journal of Public Health, 109*(3), 327-337. https://doi.org/10.17269/s41997-018-0070-5

Hrabok, M., Delorme, A., & Agyapong, V. I. O. (2020). Threats to mental health and well-being associated with climate change. *Journal of Anxiety Disorders, 76*, 1-5. https://doi.org/10.1016/j.janxdis.2020.102295

Domain 3: Social interactions about climate change

Galway, L. P., Beery, T., Buse, C., & Gislason, M. K. (2021). What Drives Climate Action in Canada's Provincial North? Exploring the Role of Connectedness to Nature, Climate Worry, and Talking with Friends and Family. *Climate, 9*(10), 1-19. https://doi.org/10.3390/cli9100146

Hickman, C. (2020). We need to (find a way to) talk about . eco-anxiety. *Journal of Social Work Practice, 34*(4), 411-424. https://doi.org/10.1080/02650533.2020.1844166

Hoggett, P., & Randall, R. (2018). Engaging with climate change: Comparing the cultures of science and activism. *Environmental Values, 27*(3), 223-243. https://doi.org/10.3197/096327118X15217309300813

Domain 4: Sustainable behaviours and climate action (negatively linked)

Galway, L. P., Beery, T., Buse, C., & Gislason, M. K. (2021). What Drives Climate Action in Canada's Provincial North? Exploring the Role of Connectedness to Nature, Climate Worry, and Talking with Friends and Family. *Climate, 9*(10), 1-19. https://doi.org/10.3390/cli9100146

Godden, N. J., Farrant, B. M., Yallup Farrant, J., Heyink, E., Carot Collins, E., Burgemeister, B., Tabeshfar, M., Barrow, J., West, M., Kieft, J., Rothwell, M., Leviston, Z., Bailey, S., Blaise, M., & Cooper, T. (2021). Climate change, activism, and supporting the mental health of children

and young people: Perspectives from western Australia. *Journal of Paediatrics and Child Health, 57*(11), 1759-1764. https://doi.org/10.1111/jpc.15649

Lawson, D. F., Stevenson, K. T., Peterson, M. N., Carrier, S. J., Seekamp, E., & Strnad, R. (2019). Evaluating climate change behaviors and concern in the family context. *Environmental Education Research, 25*(5), 678-690. https://doi.org/10.1080/13504622.2018.1564248

Verplanken, B., & Roy, D. (2013). "My worries are rational, climate change is not": Habitual ecological worrying is an adaptive response. *PloS One, 8*(9), 1-6. https://doi.org/10.1371/journal.pone.0074708

1. First collection of possible situations based on these four domains (03/11/2021)

Situations likely to induce climate anxiety

Media (9)

- News about a climate catastrophe
- News about problematic changes in temperature and weather
- News about environmental pollution
- News about government's climate action plan not being achieved
- News about climate activism
- News about climate change deniers
- Ads for disposable products
- Ads for animal products
- Influencers living and promoting highly pollutive lifestyles

Witnessing pollution in "real life" (8)

- Seeing litter in the street/park/forest
- Witnessing someone litter
- Seeing or smelling CO_2 emissions from cars
- Seeing or smelling emissions from industrial plants
- Seeing someone leaving the lights on when leaving a room
- Seeing someone leaving the water running
- Seeing someone waste food
- Seeing someone leaving their car running when parked

Social interactions (2)

- Getting into an argument with a "climate change denier"
- Talking with someone about climate change consequences

Own behaviour that is not conform with pro-environmental values (13)

- Purchasing more food than needed/intended
- Buying exotic fruit/veg from distant sources
- Shopping online instead of shopping locally
- Throwing away still edible food
- Eating animal products
- Ordering take-away
- Purchasing plastic wrapped items
- Using disposable cutlery, cups, or straws

- Using disposable hygiene products instead of reusable ones
- Using disposable face masks instead of reusable ones
- Buying fast fashion
- Taking the car instead of the train/bike/walking
- Travelling by jet

Situations likely to reduce climate anxiety

Media (5)

- Tips on sustainable behaviour
- News/documentaries on advances in green energy production
- Promotion of second-hand fashion (by influencers, peers, ads)
- Promotion of free-from products (by influencers, peers, ads)
- Ads for free-from products
- Promotion of home-grown fruit/veg
- Ads for locally sourced products

Witnessing pro-environmental behaviour (4)

- Seeing someone using reusable shopping bags
- Seeing someone using reusable cutlery, cups, or straws
- Seeing pro-environmental action in your community (e.g., a school establishing a school garden)
- Overhearing constructive discussion about climate change

Own behaviour conform with pro-environmental values (7)

- Recycling
- Using reusable drinking bottles
- Eating plant-based products
- Buying unwrapped fruit/veg
- Using paper bags or reusable bags instead of plastic bags
- Walking/cycling/taking train instead of car
- Pro-environmental investments (e.g., electric car, solar panels, environmental companies)
- Donations to pro-environmental organisations (e.g., Greenpeace, WWF)
- Engaging in climate activism
- Carpooling
- Being out in unpolluted nature

2. Revision of initial situations to reduce overlap of similar situations (12/11/2021)

Media

- Hearing about a climate catastrophe on the news (e.g., flooding, earth quakes)
- Seeing ads for disposable products (e.g., disposable razors)
- Hearing about climate activists on the news*
- Hearing about the government's climate action plan not being achieved
- Watching animal/ nature documentaries
- Seeing ads for animal products

- Seeing influencers promote environmentally unsustainable lifestyles on social media (e.g., using private jets, ‘fashion hauls’)
- Seeing news on advances in green energy production*
- Seeing ads for locally sourced products*
- Hearing about climate protests that I did not attend

Witnessing pollution in “real life”

- Seeing litter in the street
- Smelling CO2 emissions (e.g., when a car drives by)
- Seeing someone turn off the lights when leaving a room*
- Seeing someone throw away food left-overs
- Seeing someone leave the water running
- Seeing someone leave their car running while parked
- Seeing pro-environmental action in your community (e.g., a school establishing a school garden)*

Interactions

- Talking about the consequences of climate change to someone who doesn’t believe in it
- Talking to someone who is giving up enjoyment (e.g., not eating meat) to be ‘green’*

Own behaviours

- Ordering take-away
- Taking the car when I have a choice not to
- Using disposable cups when I have a choice not to
- Purchasing items wrapped in plastic
- Choosing to eat plant-based products instead of animal products*
- Buying reusable hygiene products and cleaning products (e.g., reusable sponges)*
- Shopping online
- Buying exotic fruit and vegetables
- Buying clothes second-hand*
- Purchasing more food than intended
- Traveling by aeroplane
- Recycling according to guidelines*
- Bringing reusable bags when shopping*
- Engaging in climate activism (e.g., attending protests, sharing posts)*
- Being in unpolluted nature*

3. Revision of situations to reduce overlap and remove less relevant situations (02/02/2022)

Media

- Hearing about a climate catastrophe on the news (e.g., flooding, earth quakes).
- Seeing ads for animal products or single-use items.
- Hearing about climate activism on the news.*
- Hearing news about the government’s climate action plan not being achieved.
- Watching animal/ nature documentaries.
- Seeing influencers promote environmentally unsustainable lifestyles on social media (e.g., using private jets, ‘fashion hauls’).

- Seeing news on advances in green energy production.*
- Seeing ads for locally sourced products.*

Witnessing pollution in ‘real life’

- Seeing litter in the street.
- Smelling CO_2 emissions (e.g., when a car drives by).
- Seeing someone turn off the lights when leaving a room.*
- Seeing someone throw away food left-overs.
- Seeing someone leave the water running/
- Seeing someone leave their car running while parked.
- Seeing pro-environmental action in your community (e.g., a school planting trees).

Interactions

- Talking about climate change to someone who doesn’t believe in it.
- Talking with someone who is giving up some kind of pleasure to be ‘green’ (e.g., giving up eating meat).*

Own behaviours

- Ordering take-away that is delivered in non-recyclable containers.
- Taking the car when I have a choice not to.
- Using disposable cups when I have a choice not to.
- Purchasing items wrapped in plastic.
- Choosing to eat plant-based products instead of animal products.*
- Buying reusable hygiene and cleaning products (e.g., washable sponges).*
- Buying exotic fruit and vegetable.
- Buying clothes second-hand.*
- Purchasing more food than intended.
- Traveling by aeroplane.
- Recycling according to guidelines.*
- Bringing reusable bags when shopping.*
- Engaging in climate activism (e.g., attending protests, sharing posts).*
- Being out in nature.*

4. Final revision of situations based on colleague discussions (08/02/2022)

Media

- Hearing about a climate catastrophe on the news (e.g., flooding, wildfires).
- Seeing ads for animal products or single-use items.
- Hearing about climate activism on the news.*
- Hearing news about the government’s climate action plan not being achieved.
- Watching animal/ nature documentaries.
- Seeing influencers promote environmentally unsustainable lifestyles on social media (e.g., using private jets, ‘fashion hauls’).
- Seeing news on advances in green energy production.*
- Seeing ads for locally sourced products.*

Witnessing pollution in ‘real life’

- Seeing litter in the street.
- Perceiving air pollution caused by fossil fuels (e.g., when a car drives by).
- Seeing someone throw away food left-overs.
- Seeing someone leave the water running.
- Seeing someone leave their car running while parked.
- Seeing pro-environmental action in your community (e.g., a school planning trees).*

Interactions

- Talking about climate change to someone who doesn't believe in it.
- Talking with someone who is giving up some kind of pleasure to be 'green' (e.g., giving up eating meat).*

Own behaviours

- Ordering take-away that is delivered in non-recyclable containers.
- Taking the car when I have a choice not to.
- Using disposable cups when I have a choice not to.
- Purchasing items wrapped in plastic.
- Choosing to eat plant-based products instead of animal products.*
- Buying reusable hygiene and cleaning products (e.g., washable sponges).*
- Buying clothes second-hand.*
- Purchasing more food than intended.
- Travelling by aeroplane.
- Recycling according to guidelines.*
- Bringing reusable bags when shopping.*
- Engaging in climate activism (e.g., attending protests, sharing posts online).*
- Visiting a loved place in nature.*

Future

- Thinking about my children's future.
- Thinking about my future.
